# Diversity and evolutionary history of RNA viruses among different horseshoe crab species

**DOI:** 10.1128/jvi.00164-25

**Published:** 2025-06-20

**Authors:** Yu-Hua Qi, Zhuang-Xin Ye, Ke-Hui Feng, Xiao-Wan Ma, Chuan-Xi Zhang, Meng-Hong Hu, Mang Shi, Jian-Ping Chen, Jun-Min Li

**Affiliations:** 1 State Key Laboratory for Managing Biotic and Chemical Threats to the Quality and Safety of Agro-products, Key Laboratory of Biotechnology in Plant Protection of Ministry of Agriculture and Zhejiang Province, Institute of Plant Virology, Ningbo University 47862, Ningbo, China; 2 Key Laboratory of Tropical Marine Ecosystem and Bioresource, Ministry of Natural Resources, Fourth Institute of Oceanography 635955 , Beihai, China; 3 International Research Center for Marine Biosciences, Ministry of Science and Technology, Shanghai Ocean University 74595, Shanghai, China; 4 Marine Biomedical Science and Technology Innovation Platform of Lin-gang Special Area, Shanghai, China; 5 The Centre for Infection and Immunity Studies, School of Science, Shenzhen Campus of Sun Yat-sen University 660329 , Shenzhen, China; 6 State Key Laboratory for Biocontrol, School of Life Sciences, Sun Yat-Sen University 26469 , Guangzhou, China; Wageningen University & Research, Wageningen, Netherlands

**Keywords:** RNA virome, horseshoe crabs, endogenous viral elements, virus-host coevolution

## Abstract

Horseshoe crabs (Xiphosura: Limulidae) are the sole surviving species of the class Merostomata, with only four extant species remaining today. Recent advances in metagenomic next-generation sequencing have unveiled a vast diversity of RNA viruses and non-retroviral endogenous RNA viral elements (nrEVEs) in invertebrates. This raises intriguing questions about the RNA virome and nrEVEs in horseshoe crabs as “living fossils,” potentially offering insights into the evolutionary relationships between RNA viruses and these ancient organisms. In this study, 22 novel RNA viruses were identified across the four horseshoe crab species by screening 117 data sets, including picornaviruses, totiviruses, a flavivirus, a rhabdovirus, as well as a plant-associated tombusvirus and a fungi-associated narnavirus. Additionally, 20 nrEVEs were identified in the genomes of the four horseshoe crab species (hcEVEs), with most sharing homology with the viral family *Chuviridae* (*N* = 11), supporting the hypothesis that modern negative-sense RNA viruses may trace their origins to ancient oceanic chuviruses. A time-scaled phylogenetic tree based on hcEVEs suggests that at least two independent ancient chuvirus infections and genome integration events occurred in the common ancestor of horseshoe crab species. Interestingly, transcriptional analyses indicated that hcEVE-containing transcripts display typical exon-intron structures in the three Asian horseshoe crab species, suggesting that these hcEVEs may have been co-opted by horseshoe crabs during coevolution. These findings advance our understanding of the RNA viruses associated with horseshoe crabs and shed light on the potential role of RNA viruses in shaping the evolutionary history of this “living fossil” arthropod host.

**IMPORTANCE:**

Recent studies have discovered abundant RNA viruses in invertebrates, revealing that viral genomes may integrate into host genomes, creating a genetic record of past infections. In this study, we explored the evolutionary relationship between RNA viruses and the four extant horseshoe crab species—the last representatives of the class Merostomata, often termed “living fossils”—by analyzing viral sequences embedded in their genomes. The presence of chuvirus-like sequences in the genomes of these horseshoe crabs suggests that modern negative-sense RNA viruses may trace their origins back to ancient chuviruses from the ocean. Furthermore, we identified at least two independent ancient integrations of chuviruses in the evolutionary history of horseshoe crabs, with one orthologous gene containing a chuvirus-derived G protein gene/coding sequence potentially inherited from a common ancestor of the three Asian species before their divergence. Our findings contribute to a deeper understanding of the long-term coevolution between RNA viruses and their arthropod hosts.

## INTRODUCTION

Horseshoe crabs belong to the Subphylum Chelicerata, Class Merostomata. Although they resemble crabs in appearance, horseshoe crabs are more closely related to arachnids such as spiders, scorpions, and ticks rather than “true” crabs (1). Their fossil relatives date back to the Late Ordovician Period (about 445 million years ago), and their modern body structure has remained largely unchanged since the Jurassic Period (200–146 million years ago) (2). Therefore, horseshoe crabs are often referred to as “living fossils.” Currently, there are only four extant species of horseshoe crabs belonging to three genera within two subfamilies distributed in the narrow sea area of eastern North America and east and southeast Asia. Three species (*Tachypleus tridentatus*, *Carcinoscorpius rotundicauda*, and *Tachypleus gigas*) inhabit the Indo-Pacific region, while the fourth horseshoe crab species (*Limulus polyphemus*) is distributed along the Atlantic coast of North America and the Gulf of Mexico (3–5). Recent phylogenetic analyses indicate that the Asian horseshoe crabs (*C. rotundicauda*, *T. tridentatus*, and *T. gigas*) form a monophyletic clade, distinct from the American species (*L. polyphemus*). However, the relationships among the Asian species remain unresolved, with some studies supporting varying sister relationships (4, 6, 7). Whole-genome analyses suggest that *C. rotundicauda* is a sister to the *Tachypleus* clade, while *L. polyphemus* remains separate from the Asian group, exhibiting a slower molecular evolutionary rate compared to other chelicerates (8).

Horseshoe crab blood is highly valuable in the biomedical industry for its unique sensitivity in detecting bacterial endotoxin (9). It has been widely utilized for decades to detect bacterial contamination in vaccines and injectable medications (10, 11). However, horseshoe crab populations have experienced a significant decline resulting from various anthropogenic factors, including habitat loss, overharvesting, marine pollution, and other human activities (12, 13). All four horseshoe crab species are listed on the International Union for Conservation of Nature as threatened species (https://www.iucnredlist.org/). Specifically, *T. tridentatus* is categorized as “Endangered” and *L. polyphemus* as “Vulnerable,” while *T. gigas* and *C. rotundicauda* are classified as “Data Deficient.” However, regional studies indicate that the populations of the latter two species are also in decline (8, 14).

Previous research on horseshoe crabs has predominantly focused on their biochemistry, immunity, medical application, ecology, and conservation (15). However, studies on the microbiome of horseshoe crabs have been limited, primarily focusing on bacteria using 16S rRNA gene amplicon sequencing (16, 17). Over the past decade, the advent of metagenomic next-generation sequencing (mNGS) has enabled large-scale virome screening from various hosts, revealing an enormous diversity of viruses. While extensive exploration of RNA virus diversity has been conducted in invertebrates, particularly arthropods (18–21), the hidden diversity of RNA viruses remains vast. The virome of horseshoe crabs, in particular, is poorly understood and has only been reported during a large-scale virome screening study of invertebrates using RNA-seq with mixed samples collected from Beihai, China (19). Therefore, the host specificity of these identified viruses remains unclear, and the unexplored viromes of the horseshoe crab species need further investigation.

During the long-term process of evolution, viruses have engaged in intricate interactions with their hosts. The entire or partial genome of the viruses occasionally integrates into the host genome and is stably inherited, resulting in the formation of endogenous viral elements (EVEs), which serve as a partial record of past viral infections (22–24). Recent studies have revealed that in addition to DNA and retroviral RNA viruses, non-retroviral RNA viruses could also be endogenized into the host genomes, forming non-retroviral endogenous RNA viral elements (nrEVEs) (24–27). Annotating and analyzing these nrEVEs can shed light on the ancestry of viral families, virus-host ranges, and ancient interactions between RNA viruses and hosts (28). The nrEVEs of horseshoe crabs were only preliminarily reported for *L. polyphemus* in an EVE survey study of 48 arthropod genomes (29). Therefore, it is important to comprehensively investigate the nrEVEs within the genomes of the current four living horseshoe crabs, which might provide essential clues to reconstruct the evolutionary history between RNA viruses and these species.

In this study, we performed an extensive analysis of RNA viromes using publicly available RNA-seq data sets of the four horseshoe crab species. Additionally, we also examined the presence of nrEVEs across the genomes of these species to trace the long-term coevolution of RNA viruses within horseshoe crabs.

## RESULTS

### Diversity of RNA viromes discovered in horseshoe crabs

**Fig 1:**
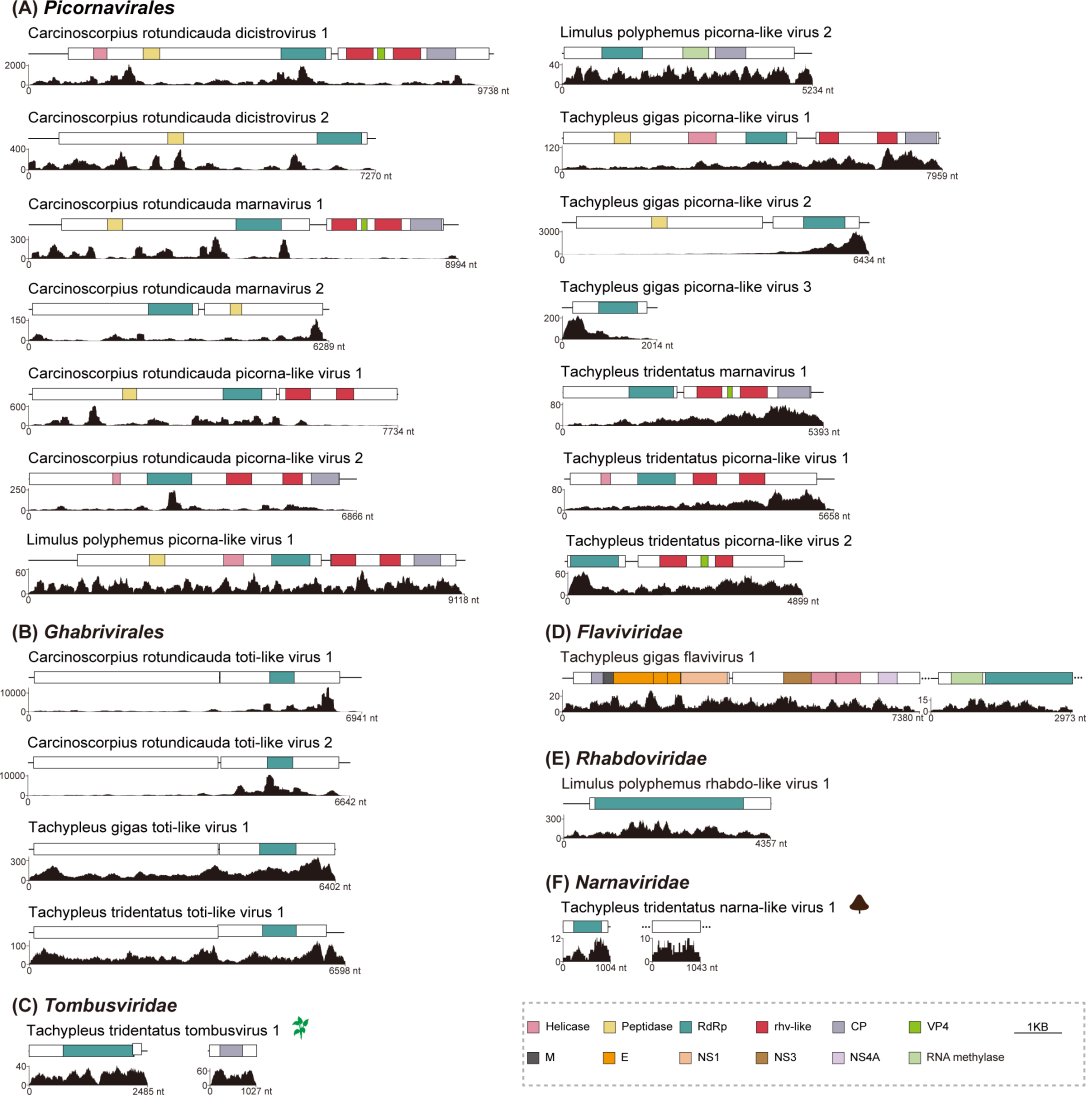
Genomic structures of novel RNA viruses identified in the four horseshoe crab species. The genomic structures of viruses identified in this study. These viruses were taxonomically classified into six groups, as depicted in panels (A) (*Picornavirales*), (B) (*Ghabrivirales*), (C) (*Tombusviridae*), (D) (*Flaviviridae*), (E) (*Rhabdoviridae*), and (F) (*Narnaviridae*). Conserved functional domains are color-coded, with domain names indicated at the bottom of the figure. CP, coat protein; E, envelope protein; M, membrane protein; NS1, NS3, and NS4A, non-structural proteins; Peptidase, peptidase C3 superfamily domain; RdRp, RNA-dependent RNA polymerase; rhv-like, picornavirus capsid-protein-domain-like; VP4, minor capsid proteins. Read coverages of the identified viruses are exhibited below the diagrams.

To investigate the RNA virome in the four horseshoe crab species, assembled contigs from 117 data sets were searched against a locally generated viral protein database. This analysis identified 22 novel RNA viruses with complete RNA-dependent RNA polymerase (RdRp) domains across 13 data sets from the four horseshoe crab species: *L. polyphemus* (*N* = 3), *C. rotundicauda* (*N* = 8), *T. tridentatus* (*N* = 6), and *T. gigas* (*N* = 5) (Table S1). The 22 newly discovered RNA viruses were closely related to the following orders/families based on their taxonomy and closest reference viruses: *Picornavirales* (*N* = 14), *Ghabrivirales* (*N* = 4), *Tombusviridae* (*N* = 1), *Flaviviridae* (*N* = 1), *Rhabdoviridae* (*N* = 1), and *Narnaviridae* (*N* = 1) (Fig. 1). Among the newly identified RNA viruses, a total of 14 picornaviruses were found in four horseshoe crab species, belonging to the families *Dicistroviridae* (Carcinoscorpius rotundicauda dicistrovirus 1&2, CrDicV1&2) and *Marnaviridae* (Carcinoscorpius rotundicauda marnavirus 1&2, CrMarV1&2; Tachypleus tridentatus marnavirus 1, TtMarV1), or unclassified picornaviruses (Carcinoscorpius rotundicauda picorna-like virus 1&2, CrPicLV1&2; Limulus polyphemus picorna-like virus 1&2, LpPicLV1&2; Tachypleus gigas picorna-like virus 1, 2, and 3, TgPicLV1, 2, and 3; Tachypleus tridentatus picorna-like virus 1&2, TtPicLV1&2) (Fig. 1A). In addition to *L. polyphemus*, four toti-like viruses (Carcinoscorpius rotundicauda toti-like virus 1&2, CrTotLV1&2; Tachypleus gigas toti-like virus 1, TgTotLV1; Tachypleus tridentatus toti-like virus 1, TtTotLV1) were identified in the other three horseshoe crab species (Fig. 1B). Interestingly, we also identified a plant-associated virus with two segments from the family *Tombusviridae* (Tachypleus tridentatus tombusvirus 1, TtTomV1) in *T. tridentatus* (Fig. 1C), a flavivirus (Tachypleus gigas flavivirus 1, TgFlaV1) with two discontinuous fragments in *T. gigas* (Fig. 1D), a rhabdo-like virus (Limulus polyphemus rhabdo-like virus 1, LpRhaLV1) in *L. polyphemus* (Fig. 1E), and a narna-like virus (Tachypleus tridentatus narna-like virus 1, TtNarLV1) in *T. tridentatus* (Fig. 1F). To further assess viral abundance and distribution across different samples, quality-controlled raw reads from each data set were mapped back to the corresponding horseshoe crab RNA viruses. The results revealed that most identified viruses were detected in only a single sample or in samples submitted by the same institution (Fig. S1A), suggesting that some viruses may exhibit geographically restricted distribution patterns. The viral contigs from the laboratory samples were confirmed using reverse transcription-PCR (RT-PCR), followed by Sanger sequencing (Fig. S1B). Detailed descriptions of these novel viruses were provided in Table S2.

### Phylogenetic analysis of novel viruses in horseshoe crabs

**Fig 2:**
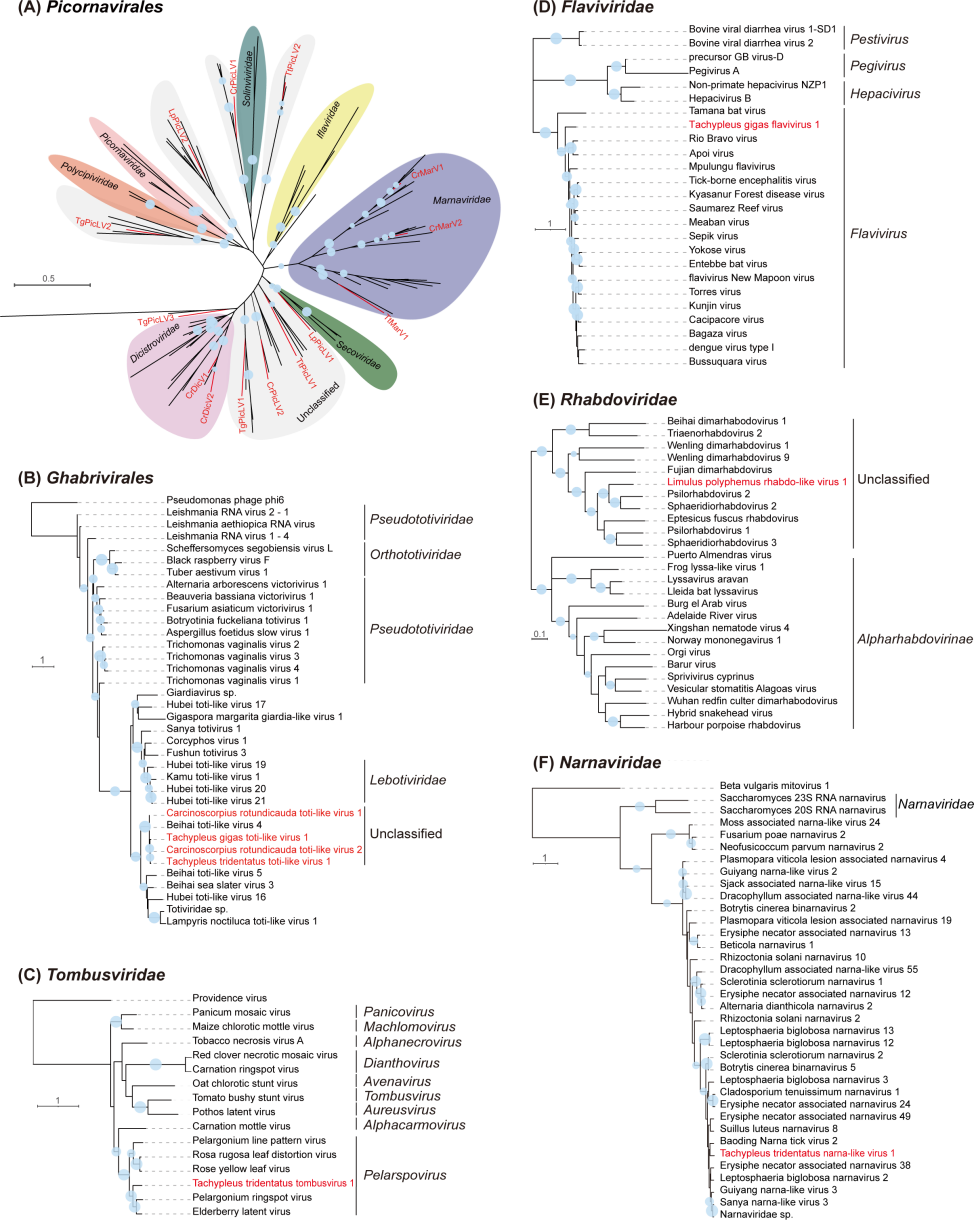
Phylogenetic trees of novel RNA viruses identified in the four horseshoe crab species. Phylogenetic trees for *Picornavirales* (**A**), *Ghabrivirales* (**B**), *Tombusviridae* (**C**), *Flaviviridae* (**D**), *Rhabdoviridae* (**E**), and *Narnaviridae* (**F**) were constructed using the maximum likelihood method based on conserved viral RdRp domains with 1,000 bootstrap replicates. The novel viruses identified in this study are highlighted in red font. Nodes with bootstrap values > 50% are marked with solid blue circles, and the larger circles indicate higher bootstrap values. The accession numbers of viruses used in this study were listed in File S2.

The phylogenetic analysis reveals the taxonomic associations between the newly discovered horseshoe crab viruses and related reference viruses (Fig. 2). Based on the phylogenetic tree, 5 of the 14 identified novel viruses in the order *Picornavirales* can be assigned into their respective families: 3 viruses in the family *Marnaviridae* (CrMarV1&2 and TtMarV1) and 2 viruses in the family *Dicistroviridae* (CrDicV1&2). The remaining nine picorna-associated viruses (CrPicLV1&2, LpPicLV1&2, TgPicLV1, 2, and 3, and TtPicLV1&2) were placed in unclassified clades (Fig. 2A). Interestingly, four novel toti-like viruses from horseshoe crabs (CrTotLV1&2, TgTotLV1, and TtTotLV1) clustered with a previously reported horseshoe crab virus, Beihai toti-like virus 4 (accession: NC_032841.1). The RdRp aa sequence similarity among these viruses ranged from 75.37% to 88.84%, suggesting a close evolutionary relationship among these toti-like viruses found in horseshoe crabs, which likely form an unclassified lineage in the order *Ghabrivirales* (Fig. 2B). Unexpectedly, a plant tombusvirus (TtTomV1) was identified in the hemocytes of *T. tridentatus*, grouping with two other plant viruses, Pelargonium ringspot virus (65.81% RdRp aa identity) and Elderberry latent virus (66.76% RdRp aa identity), in the genus *Pelarspovirus* of the family *Tombusviridae* (Fig. 2C). While it is unusual for a plant tombusvirus to directly infect horseshoe crabs, the presence of TtTomV1 in the hemocyte samples may result from external contamination during sample collection or from dietary intake of plant-virus-associated materials. Additionally, a novel flavivirus (TgFlaV1) of *T. gigas* was distantly clustered with other members of the genus *Flavivirus* (Fig. 2D). A rhabdovirus (LpRhaLV1) identified in *L. polyphemus* clustered with two trematode parasite-associated rhabdoviruses (sharing 64.12% and 62.36% aa identities, respectively) within an unclassified lineage of the family *Rhabdoviridae* (Fig. 2E). Furthermore, a novel narnavirus (TtNarLV1) with two separate contigs, identified from whole-body samples of *T. tridentatus*, clustered with an unclassified lineage likely associated with fungi in the family *Narnaviridae* (Fig. 2F). This implies that the virus might originate from fungi associated with *T. tridentatus*, such as symbiotic or parasitic fungi in its habitat or body.

### nrEVEs identified in the genomes of the four horseshoe crab species

**Fig 3:**
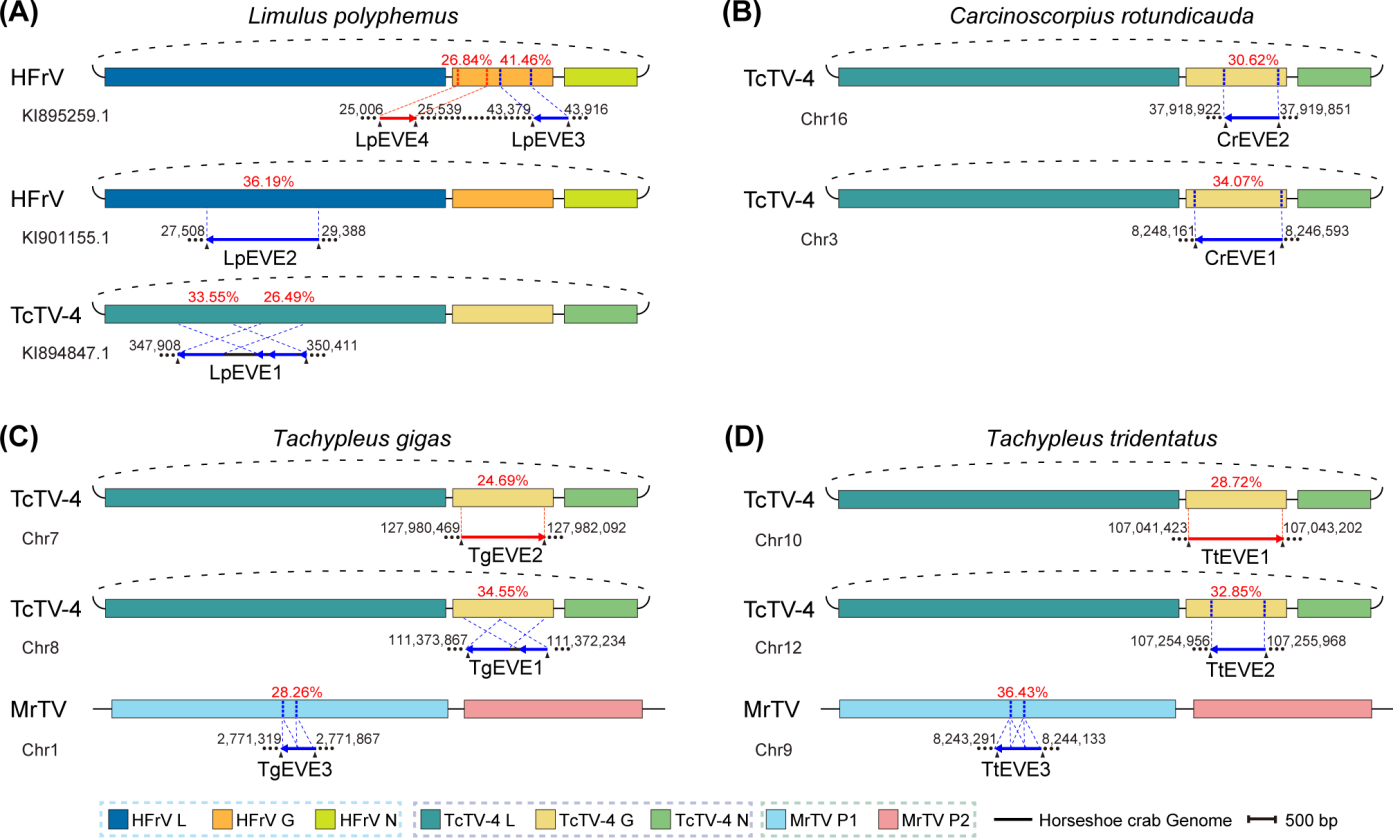
Non-retroviral endogenous viral elements (nrEVEs) identified in the genomes of four horseshoe crab species. The schematic diagram illustrates the identified nrEVEs (longer than 500 nt) within the genomes of *Limulus polyphemus* (**A**), *Carcinoscorpius rotundicauda* (**B**), *Tachypleus gigas* (**C**), and *Tachypleus tridentatus* (**D**). Each nrEVE is associated with the corresponding exogenous virus with the highest similarity. Specific homology regions between nrEVEs and their corresponding exogenous viruses are indicated by blue dotted lines, with percentage amino acid identities highlighted in red font. nrEVEs that align in the same direction with the genome sequence are represented by red lines, while blue lines depict the opposite direction. G, glycoprotein; HFrV, Herr Frank virus 1; L, RNA-dependent RNA polymerase; MrTV, *Macrobrachium rosenbergii* Taihu virus; N, nucleoprotein; P1, non-structural polyprotein; P2, capsid protein precursor; TcTV-4, Tacheng Tick Virus 4.

To systematically identify nrEVEs in horseshoe crab genomes (hcEVEs), a tBlastN search was conducted using non-retroviral RNA viruses in the kingdom *Orthornavirae* against the genomes of the four horseshoe crab species. The analysis revealed the presence of six hcEVEs in *L. polyphemus* (LpEVE1–6, lengths 282–2,504 nt), five in *C. rotundicauda* (CrEVE1–5, lengths 261–1,569 nt), four in *T. gigas* (TgEVE1–4, lengths 342–1,634 nt), and five in *T. tridentatus* (TtEVE1–5, lengths 336–1,780 nt) (Table S3). The existence of TtEVE1–5 was further confirmed by PCR, followed by Sanger sequencing (Fig. S1C). These hcEVEs (longer than 500 nt) were integrated into the host genomes in either the forward or reverse direction and showed homology to the viral structural and non-structural proteins (Fig. 3). Specifically, hcEVEs related to the exogenous viruses from the family *Chuviridae* (Tacheng Tick Virus 4, TcTV-4; Herr Frank virus 1, HFrV) were identified in all four horseshoe crab genomes. Notably, LpEVE1 and LpEVE2 correspond to the L protein of TcTV-4 and HFrV, respectively (Fig. 3A), while all chu-related nrEVEs in the other three horseshoe crab species exclusively exhibit homology to the TcTV-4 G protein (Fig. 3B through D). Although no exogenous chuviruses were identified in this study, the widespread integration of the chuvirus-related viral sequences in the horseshoe crab genomes suggests a long-term coexistence of chuviruses with horseshoe crab hosts. Additionally, hcEVEs (TgEVE3 and TtEVE3) showing homology with the non-structural polyprotein (P1) of Macrobrachium rosenbergii Taihu virus (MrTV, family *Dicistroviridae*) were found in the genomes of *T. gigas* and *T. tridentatus*, respectively (Fig. 3C and D).

### Phylogenetic analysis of hcEVEs reveals the evolutionary history of ancient chuvirus infections in horseshoe crabs

**Fig 4:**
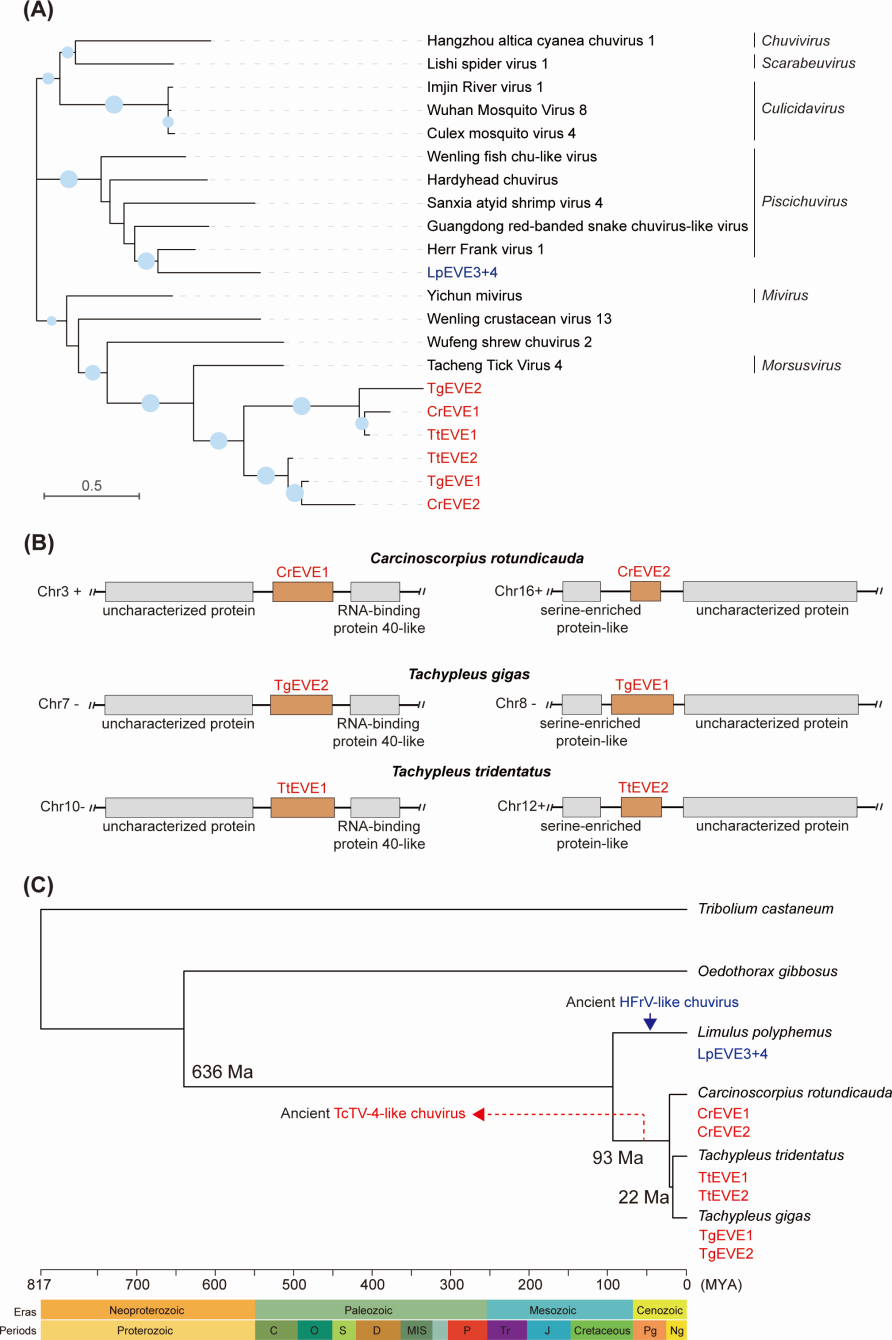
Construction of ancient infection histories of chuviruses in the four horseshoe crab species. (**A**) Phylogenetic analysis of horseshoe crab nrEVEs and related exogenous viruses based on chuvirus glycoprotein sequences. Nodes with bootstrap values > 50% are marked with solid blue circles, and the larger circles indicate higher bootstrap values. (**B**) Identification of orthologous hcEVEs in horseshoe crab genomes. Orthologs of the integrations were established based on the syntenic arrangement of their most-proximal genes. (**C**) Reconstruction of time-scaled phylogenetic tree based on the genomes of the four horseshoe crabs and associated arthropod species (*Tribolium castaneum* and *Oedothorax gibbosus*). Non-retroviral endogenous viral elements of the four horseshoe crab species (hcEVEs) are highlighted in red font. Arrows indicate potential ancient chuvirus integration events: TcTV-4 (red) and HFrV (blue). HFrV, Herr Frank virus 1; TcTV-4, Tacheng tick virus 4.

Ancient viral integration events into host genomes can be inferred by examining the divergence time of the hosts and the orthologous relationships among EVEs (30, 31). Given that hcEVEs derived from the G protein of chuviruses (TcTV-4 and HFrV) were identified in all four horseshoe crab species (Fig. 3), we constructed a phylogenetic tree based on the G proteins to investigate the relationship between hcEVEs and their corresponding exogenous chuviruses. The results revealed that six hcEVEs from three Asian horseshoe crab species (*C. rotundicauda*, *T. gigas*, and *T. tridentatus*) were separated into two distinct groups and clustered together with the G protein of TcTV-4 (genus *Morsusvirus*) (Fig. 4A). Interestingly, alignment of the six hcEVEs suggested that these two groups can also be distinguished by specific regions, notably at the nucleotide positions of 138-151 and 294-322 (Fig. S2A). Pairwise distance analysis also demonstrated high nucleotide identities within each group (Fig. S2B). In contrast, hcEVEs of *L. polyphemus* (LpEVE3+4) were grouped with virus members of a different genus, *Piscichuvirus* (Fig. 4A), suggesting a distinct endogenization event for this species. Notably, each of the three horseshoe crab species (*C. rotundicauda*, *T. gigas*, and *T. tridentatus*) contains hcEVEs belonging to distinct groups in the phylogenetic tree (Fig. 4A). This suggests that each hcEVE group may have been inherited from a corresponding hcEVE in a common ancestor of the horseshoe crab species. Additionally, the two groups likely originated from a duplication event following the endogenization of a single chuvirus infection in the ancestral species.

To distinguish between orthologous and non-orthologous integrations, we first examined the genomic positions of the six hcEVEs derived from the G protein gene/coding sequence of chuviruses in the horseshoe crab genomes. As shown in Fig. 4B, each hcEVE group was located in the same genomic locus across all three horseshoe crab species and shared two identical adjacent ORFs. Notably, these EVE sequences were absent in the *L. polyphemus* genome. The nucleotide identities of the flanking regions (2 kb) of hcEVEs exceeded 75% (Fig. S2C), indicating that the genes containing the two hcEVE groups are orthologous.

To further explore the ancient endogenization of these hcEVEs, we constructed a time-scaled phylogenetic tree based on the four horseshoe crab species. Our analysis suggests two independent viral integration events in ancient times, one happened before the divergence of three Asian horseshoe crabs, resulting in the endogenization of the TcTV-4 G protein into the genomes of *C. rotundicauda* (CrEVE1&2), *T. gigas* (TgEVE1&2), and *T. tridentatus* (TtEVE1&2). The second integration event likely occurred after the divergence of *L. polyphemus* from the other three horseshoe crab species, resulting in the presence of LpEVE3+4 within the *L. polyphemus* genome (Fig. 4C).

### Analysis of conserved domains potentially associated with hcEVE integration

To investigate the molecular mechanisms that may facilitate the integration of hcEVEs into the horseshoe crab genome, we analyzed the ±10 kb flanking regions surrounding these elements. Several putative integration-related domains were identified, including those associated with reverse transcriptase (RT), DNA transposase, and other auxiliary functions (Table S6). Notably, an RT-related domain (RT_RNaseH_2 superfamily) was found adjacent to LpEVE4. DNA transposon-related domains were detected in the flanking regions of LpEVE3 and LpEVE4, CrEVE1, TgEVE2, and TtEVE1. Additionally, GIY-YIG nuclease and DEAD-box helicase domains, both of which may contribute structurally or catalytically to the integration process, were also identified (Table S6). Interestingly, the flanking regions of CrEVE1, TgEVE2, and TtEVE1, which cluster into a distinct phylogenetic branch (Fig. 4A), harbored identical DNA transposon-related domains (Transposase_1 superfamily), supporting their putative orthologous relationship.

### Transcription profiles of hcEVEs in different populations of horseshoe crabs

**Fig 5:**
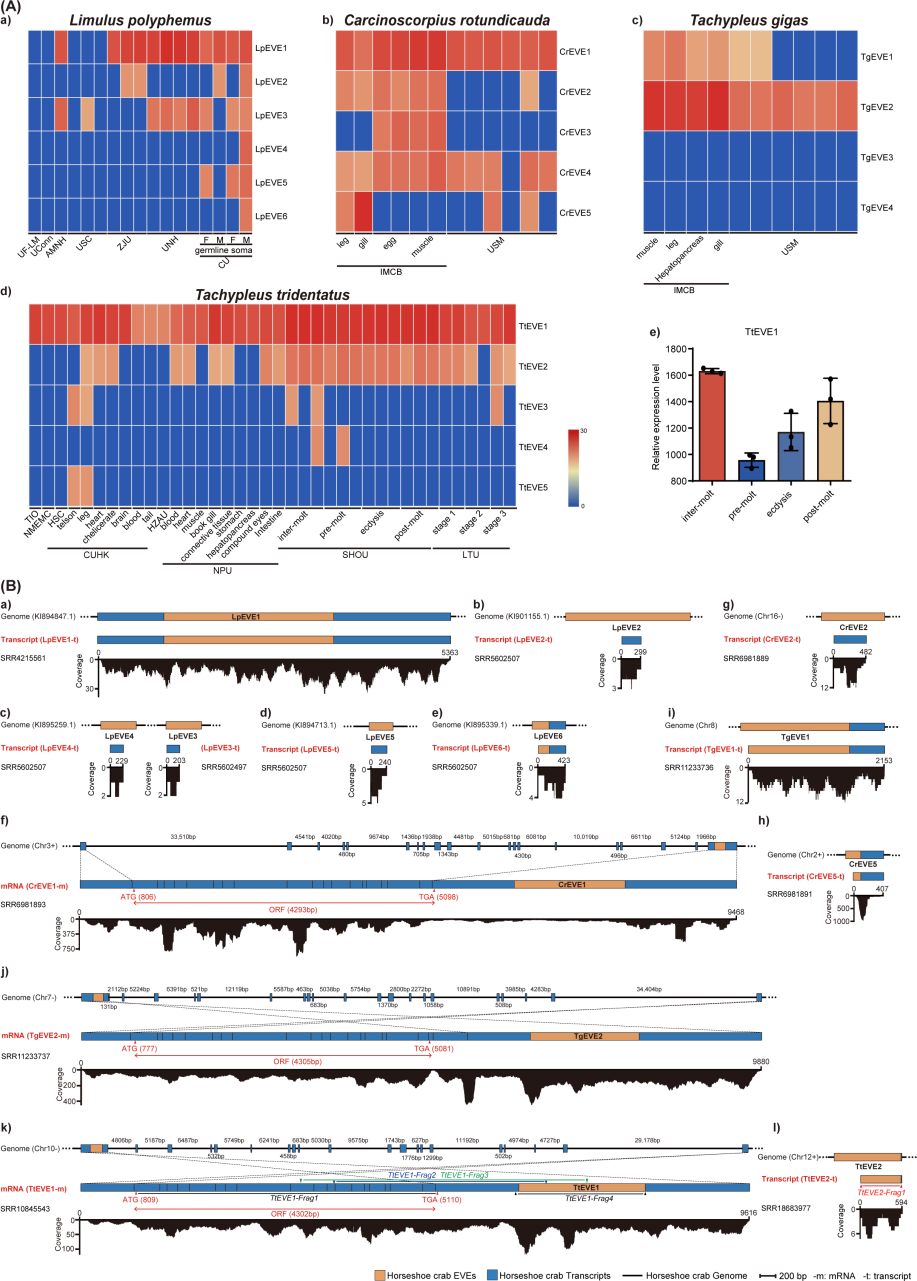
Transcription of hcEVEs in the four horseshoe crab species. (**A**) The heatmap represents the abundance of transcriptional reads derived from hcEVEs of *Limulus polyphemus*, *Carcinoscorpius rotundicauda*, *Tachypleus gigas*, and *Tachypleus tridentatus* across various horseshoe crab populations. AMNH, American Museum of Natural History; CU, University of Cambridge; CUHK, The Chinese University of Hong Kong; HZAU, Huazhong Agricultural University; IMCB, Institute of Molecular and Cell Biology; LTU, La Trobe University; NMEMC, National Marine Environment Monitoring Center; NPU, Northwestern Polytechnical University; SHOU, Shanghai Ocean University; TIO, Third Institute of Oceanography; UCONN, University of Connecticut; UF-LM, University of Florida – Leonid Moroz; UNH, University of New Hampshire; USM, Universiti Sains Malaysia; USC, University of South Carolina; ZJU, Zhejiang University. (**B**) Schematic diagrams represent the position and coverage of hcEVEs-containing transcripts within the genomes of *L. polyphemus* (a–e), *C. rotundicauda* (f–h), *T. gigas* (i, j), and *T. tridentatus* (k, l). Predicted open reading frames (ORFs) were indicated with red double-headed arrows. *TtEVE1-Frag1*, *2*, *3*, and *4* indicate the corresponding transcripts of the horseshoe crab species confirmed with RT-PCR followed by Sanger sequencing.

To investigate a comprehensive understanding of the potential transcription of the identified hcEVEs, we obtained and screened 77 publicly available horseshoe crab data sets submitted by various researchers. These data sets included 17 from *L. polyphemus*, 12 from *C. rotundicauda*, 10 from *T. gigas*, and 38 from *T. tridentatus* (Table S4). The results indicated that, except for the two hcEVEs from *T. gigas* (TgEVE3–4), all other 18 hcEVEs were detected in at least one data set from the four horseshoe crabs (Fig. 5A). Some hcEVEs, such as LpEVE1, CrEVE1, CrEVE4, TgEVE2, TtEVE1, and TtEVE2, exhibited widespread and relatively high transcript expression levels across most data sets, suggesting they may be associated with conserved biological functions in horseshoe crabs (Fig. 5A). Notably, some hcEVE transcripts were expressed exclusively in data sets from specific institutions, suggesting potential population- or condition-specific transcription patterns. For instance, LpEVE4-6 were exclusively transcribed in data sets from the University of Cambridge, while CrEVE3 was predominantly expressed in samples from the Institute of Molecular and Cell Biology (Fig. 5A–a and b). This suggests that hcEVEs transcription might be associated with specific horseshoe crab populations. Additionally, the distinguished expression profile of specific hcEVE might also be influenced by factors such as gender (LpEVE4–6), tissue types (CrEVE3, 5 and TtEVE5), and developmental stages (TtEVE2–4) of horseshoe crabs (Fig. 5A–a, b and d). While TtEVE1 was found to be ubiquitously transcribed across all four developmental stages of *T. tridentatus* submitted by Shanghai Ocean University (Fig. 5A–d), the highest expression of TtEVE1 occurred during inter-molt stages, followed by post-molt, ecdysis, and pre-molt stages (Fig. 5A–e). This might reflect its involvement in molting-related physiological processes, possibly as a regulator of transcriptional changes during molting.

### Some hcEVEs might have evolved as integral components of horseshoe crab mRNAs

To further investigate the presence of hcEVEs within host transcripts and genomes, we used hcEVE sequences as queries to search against the transcriptomes and genomes of the corresponding horseshoe crab species. The results confirmed that 13 hcEVEs were detected in the transcripts of the four horseshoe crabs: 6 in *L. polyphemus* (LpEVE1–6), 3 in *C. rotundicauda* (CrEVE1, 2, and 5), 2 in *T. gigas* (TgEVE1&2), and 2 in *T. tridentatus* (TtEVE1&2) (Fig. 5B). However, only six of these hcEVE-containing transcripts were relatively long (>600 bp) or exhibited high coverage (>20×): LpEVE1, CrEVE1, CrEVE5, TgEVE1&2, and TtEVE1. Unexpectedly, although these hcEVEs were present in the horseshoe crab transcriptomes, we failed to identify piRNAs derived from these hcEVEs in the corresponding small RNA data sets. Notably, three highly homologous hcEVEs (CrEVE1, TgEVE2, and TtEVE1) (Fig. S2B) were contained by three long transcripts displaying typical eukaryotic exon-intron structures, tentatively named CrEVE1-m (Fig. 5B–f), TgEVE2-m (Fig. 5B–j), and TtEVE1-m (Fig. 5B–k). The presence of TtEVE1-m in *T. tridentatus* was further validated using RT-PCR followed by Sanger sequencing (Fig. S1D). Given that the exon-intron structure is a hallmark of eukaryotic genes, we speculated that these hcEVEs may have been co-opted by horseshoe crabs and evolved as integral components of their mRNAs. Moreover, both the predicted ORFs (5′ terminus) and hcEVEs (3′ terminus) within the three mRNA (CrEVE1-m, TgEVE2-m, and TtEVE1-m) displayed extremely high identities exceeding 95% for the nucleotide sequences, suggesting that the genes corresponding to these mRNAs in the three horseshoe crab species may have originated from a common ancestral gene.

## DISCUSSION

Over the past decade, significant advancements in mNGS and bioinformatics tools have led to the unbiased discovery of a multitude of RNA viruses, greatly enhancing our understanding of RNA viromes and viral evolution (19, 32–34). The exploration of RNA virus diversity also leads to the identification of nrEVEs in various eukaryotes, representing an extensive fossil record for the iterative molecular armrace of defense and counter-defense strategies between RNA viruses and hosts spanning millions of years (35). In this study, the RNA virome of the “living fossils,” horseshoe crabs, was identified, and virus-host coevolution was revealed through extensive analysis of nrEVEs within the genomes of four horseshoe crab species.

A total of 22 novel RNA viruses with remarkable genomic coverage (>800×) were discovered in four horseshoe crab species, all of which are distinct from previously reported ones identified from mixed samples of *C. rotundicauda* and *T. tridentatus* (19), suggesting that RNA virus diversity in horseshoe crabs may have been significantly underestimated. The four newly discovered toti-like viruses formed a distinct unclassified clade with previously reported horseshoe crab toti-like virus (Beihai toti-like virus 4, NC_032841.1) (Fig. 2B), implying the prevalence of toti-like viruses in horseshoe crabs. Although the natural hosts of totiviruses previously approved by the International Committee for the Taxonomy of Viruses (ICTV) are protozoa and fungi (36), numerous candidate totiviruses recently discovered in insects (21, 37), crustaceans (38), and the horseshoe crabs have now been classified into several new families under the order *Ghabrivirales* according to the latest ICTV MSL40 (https://ictv.global/news/taxonomy_2024). Additionally, this study also expanded the host range of an important viral family (*Flaviviridae*) to species in the class Merostomata, as we detected a flavivirus (TgFlaV1) in *T. gigas* (Fig. 1D and 2D). Furthermore, based on the closely related viruses in the phylogenetic tree, it is presumed that the authentic hosts of the newly identified tombusvirus (TtTomV1) (Fig. 2C), rhabdo-like virus (LpRhaLV1) (Fig. 2E), and narna-like virus (TtNarLV1) (Fig. 2F) might be associated with the plant diets, parasitic trematodes, and fungi of horseshoe crab species, respectively. It should also be noted that the RNA-seq data sets used in this study were retrieved from public databases and were not specifically optimized for viral sequence enrichment (e.g., through ribosomal RNA depletion or viroid RNA purification). As a result, the true diversity and prevalence of RNA viruses in horseshoe crabs may be underestimated.

EVEs, serving as genomic fossils, offer invaluable insights into various aspects of ancient viral evolutionary history, such as host ranges, ancestral viral genetic diversity, and the dating of ancestral viral infection events (28, 39). Our study identified 20 nrEVEs in the genomes of four horseshoe crab species, and most of these hcEVEs were homologous to extant viruses of the family *Chuviridae* (*N* = 11), followed by *Dicistroviridae* (*N* = 7) and *Lispiviridae* (*N* = 2). Although only two exogenous dicistroviruses (CrDicV1&2) were identified in *C. rotundicauda*, it suggests a historical relationship between horseshoe crabs and viruses from these three families in ancient times. The previously proposed hypothesis of the evolutionary scenario suggested that the emergence of chuviruses in marine invertebrates might be the origin of extant negative RNA viruses (cytorhabdovirids) (34). Additionally, recent studies reported a diverse range of nrEVEs in insect genomes, with the most widespread and abundant ones derived from the *Rhabdoviridae* and *Chuviridae* families (40). The widespread integration of chuvirus-derived hcEVEs (Fig. 3) suggests that horseshoe crabs, as ancient marine organisms, were hosts to ancient chuvirus infections. This finding may support the hypothesis that modern negative-sense RNA viruses trace their origins back to ancient chuviruses from the ocean. Additionally, more frequent insertion of hcEVEs derived from G protein gene/coding sequences was detected compared to the L and N proteins of chuviruses, which is similar to the previous report observed for the chuvirus-derived nrEVEs in genomes of other arthropods (41). The preferential integration or retention of chuvirus G protein-derived hcEVEs may be due to the fact that the G protein gene/coding sequence is being captured by host endogenous retrotransposons, followed by intragenomic replication and amplification, as previously demonstrated in mosquitoes (41). Another possibility is that glycoproteins, being surface proteins involved in host-virus interactions, may confer selective advantages and be co-opted by the host, leading to positive selection or retention of glycoprotein-derived EVEs. In contrast, L or N proteins may offer limited or no benefit to the host and could even be deleterious, making their retention less likely.

Another benefit of the EVE study is to understand prehistoric virus-host coevolutionary history over geological timescales (24). Study of endogenous bornavirus-like elements helped to reconstruct a 100 million-year history of bornavirus infections hidden in vertebrate genomes, and analysis of pestivirus-derived nrEVEs suggested their presence as homologous sequences in two subfamilies of shrews, indicating an integration event that occurred before the last common ancestor of the subfamily, over 10.8 million years ago (30, 31). In this study, a time-scaled phylogenetic tree based on the hcEVEs suggested that ancient TcTV-4-like chuvirus infection could be traced back before the species differentiation of the three Asian horseshoe crabs (*C. rotundicauda*, *T. tridentatus*, and *T. gigas*), resulting in the integration of G protein-associated homologous hcEVEs into the genomes of these horseshoe crab species (Fig. 4B and C). This finding is consistent with previous research, consolidating a closer evolutionary relationship among the three Asian horseshoe crab species (4, 6, 7).

The identification of RT and transposase-related domains in the flanking regions of hcEVEs offers valuable insight into the possible molecular mechanisms facilitating the integration of non-retroviral RNA viruses into host genomes (28). Although such viruses inherently lack integrase activity, they may become endogenized through host-mediated processes (24). In this study, the presence of an RNase H-like RT domain near LpEVE4 suggests that viral RNA may have been reverse-transcribed into cDNA by host- or transposon-derived enzymes prior to integration (Table S6). Furthermore, DNA transposon-related domains, particularly those from the DDE_Tnp and Transposase_1 superfamilies, were found adjacent to several hcEVEs (e.g., LpEVE3 and LpEVE4, CrEVE1, TgEVE2, and TtEVE1), implying a potential role of these DNA transposases in mediating genomic insertion. Additional domains such as GIY-YIG nuclease and DEAD-box helicase, identified near certain hcEVEs, may have provided structural or catalytic support during the integration process. Notably, identical transposon-related domains in the flanking regions of three orthologous ChuEVEs (CrEVE1, TgEVE2, and TtEVE1) support the hypothesis of an ancient viral integration event predating species divergence.

Recent studies indicated that some of the nrEVEs in arthropods, especially insects, could produce corresponding transcripts, including small RNAs, non-coding RNAs, and mRNAs derived from nrEVEs (42–44). In this study, the majority of identified hcEVEs (18 out of 20) were found to be transcriptionally active based on transcriptome screening of the corresponding horseshoe crab species (Fig. 5). It was observed that the transcription of most hcEVEs was variously presented across different horseshoe crab populations (Fig. 5A). This phenomenon may be due to the highly variable distribution and polymorphism level of nrEVEs in different individuals/populations as indicated previously (45, 46), or some hcEVEs might be transcribed under certain environmental conditions. Moreover, the expression of specific hcEVEs might be associated with the gender, tissue types, or developmental stages of horseshoe crab species, suggesting potential roles of hcEVEs involved in host biological processes. Although previous studies have suggested that EVEs may serve as sources of piRNAs or siRNAs in arthropods (44, 47), our analysis of small RNA data sets from horseshoe crabs (excluding *T. gigas*, which lacks sRNA data) did not detect piRNAs or siRNAs derived from hcEVEs. This result aligns with findings from other studies, which show that nearly all EVEs in *L. polyphemus* are located outside of piRNA clusters (29). In *Aedes aegypti*, EVEs within piRNA clusters are thought to contribute to antiviral immunity (47). Additionally, it is noteworthy that the genomes of certain aphid species harboring endogenous negevirus-like elements (ENVEs) were typically free of active nege-like virus infections, suggesting that ENVEs may exert antiviral effects against their cognate viruses (48). In this study, we observed the prevalence of hcEVEs across the four horseshoe crab species, despite the absence of detectable contemporary chuviruses. Although hcEVE-derived piRNAs were not identified, it is possible that hcEVEs may mediate antiviral activity to their cognate chuviruses through mechanisms other than the piRNA pathway in horseshoe crabs. An alternative explanation is that actively replicating chuviruses may indeed exist in horseshoe crabs but were not detected due to the limited data sets analyzed in this study. Future investigations incorporating broader and more diverse sampling are expected to reveal the presence of exogenous chuviruses in horseshoe crabs.

One of the key considerations regarding EVE integration revolves around their potential impact on the host’s biology. If EVEs are detrimental to their hosts, they are likely to be rapidly eliminated from populations through allele segregation under natural selection. Neutral EVEs, on the other hand, may spread within populations through genetic drift, but they are expected to accumulate random mutations and eventually lose their identity as EVEs. EVEs that provide fitness advantages to their hosts are expected to be functionally adopted (co-opted) and spread throughout host populations, exhibiting higher sequence conservation (43, 49, 50). Although functional studies of EVEs were mostly concentrated on EVEs derived from retroviruses (ERVs) (51), recent investigations suggested that the domestication of nrEVEs has also provided numerous benefits for host biological functions, involving in host antiviral immunity and being repurposed to promote the development of novel cellular functions (29, 42, 44, 46, 47, 52). Interestingly, our analysis revealed that chuvirus-derived hcEVEs produced highly homologous transcripts with predicted intact ORFs (CrEVE1-m, TgEVE2-m, and TtEVE1-m) in three Asian horseshoe crab species (Fig. 5B–f, j and k), which is similar to the previous reports of nrEVE transcripts homologous to ﬂaviviruses and bornaviruses (26, 53). Additionally, exon-intron structures were discovered in these three hcEVE-containing transcripts, which is a hallmark of eukaryotic genes (54), implying that these hcEVEs might be tamed as authentic mRNAs of the horseshoe crabs. Although the function of these hcEVEs is currently unclear, the downstream location of the hcEVEs to the predicted ORFs (Fig. 5B–f, j and k) and high sequence identity among them (Fig. S2B) suggested that they might play a role in gene regulation inherited from ancestral horseshoe crabs.

**Fig 6:**
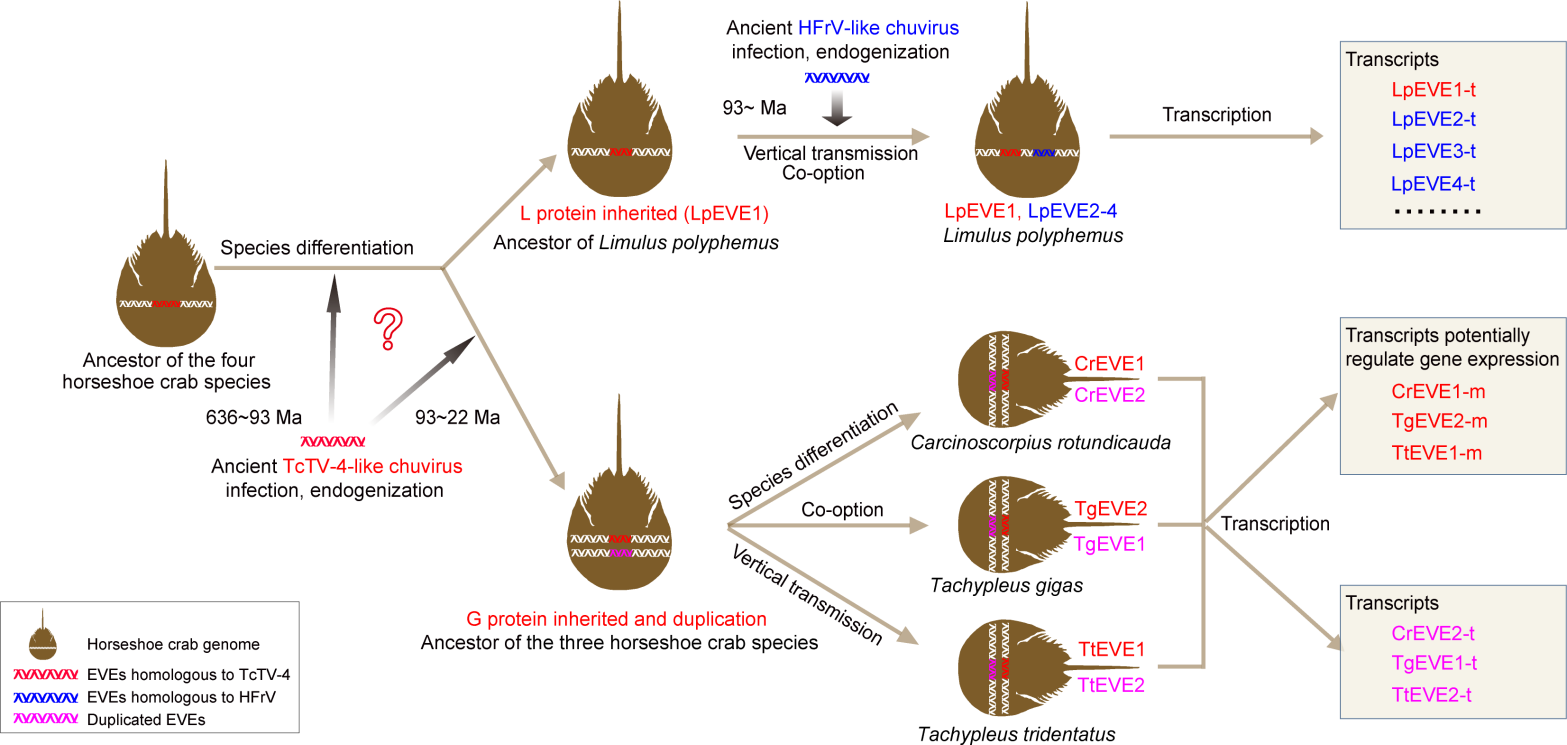
Schematic diagram illustrates the long-term coevolution between ancient chuviruses and the four horseshoe crab species. A hypothesis is proposed that at least two independent chuvirus integration events occurred in the genomes of the ancestor of horseshoe crab species. One is that an ancient TcTV-4-like chuvirus infection and subsequent endogenization event may have occurred before the last common ancestor of the three Asian horseshoe crab species, or even earlier. This would have led to the integration of viral G protein gene/coding sequences into the ancestor’s genome. It remains uncertain whether the endogenization of the viral L protein sequence in the *L. polyphemus* genome (LpEVE1) resulted from the same or another separate event. The gene containing the "G protein" in the ancestor species likely underwent duplication, forming two paralogous genes that were inherited by the three descendant Asian horseshoe crabs (*C. rotundicauda*, *T. tridentatus*, and *T. gigas*) after species differentiation. The stably inherited viral L and G-associated hcEVEs co-evolved with their hosts and were potentially domesticated into functional transcripts (LpEVE1-t, CrEVE2-t, TgEVE1-t, and TtEVE2-t) or actual genes (CrEVE1-m, TgEVE2-m, and TtEVE1-m) in the four horseshoe crab species. Another event might have occurred after the formation of *L. polyphemus* species, following infection and endogenization by ancient HFrV-like chuvirus, leading to the activated transcription of LpEVE2, 3, and 4. HFrV, Herr Frank Virus 1; TcTV-4, Tacheng Tick Virus 4.

In summary, this study has unveiled the RNA viromes of horseshoe crab species and reconstructed the long-term coevolution of RNA viruses with this ancient species through analysis of their hcEVEs. As illustrated in a schematic diagram (Fig. 6), we propose a hypothesis that an ancient TcTV-4-like chuvirus infection and subsequent endogenization event may have occurred prior to the last common ancestor of the three Asian horseshoe crab species approximately 93–22 million years ago, or even earlier, leading to the integration of viral G protein gene/coding sequences into the genome of the ancestor host. However, it remains unclear whether the endogenization of viral L protein sequence in the *L. polyphemus* genome (LpEVE1) resulted from the same ancient TcTV-4-like chuvirus infection or from an independent event. The “G protein”-containing gene underwent further duplication, possibly forming two paralogous genes and inheriting by the three descendant Asian horseshoe crabs (*C. rotundicauda*, *T. tridentatus*, and *T. gigas*) after species differentiation. The stably inherited viral L and G-associated hcEVEs co-evolved with their hosts and were potentially domesticated as functional transcripts (LpEVE1-t, CrEVE2-t, TgEVE1-t, and TtEVE2-t) or authentic genes (CrEVE1-m, TgEVE2-m, and TtEVE1-m) of the four extant species of horseshoe crabs (Fig. 6). Additionally, another EVE insertion event may have occurred after the species formation of *L. polyphemus* that was infected by another ancient HFrV-like chuvirus, bringing the active transcriptions of LpEVE2, 3, and 4. Collectively, our study will help elucidate how RNA viruses may have contributed to the evolution of this “living fossil” arthropod host.

## MATERIALS AND METHODS

### RNA-seq data set of horseshoe crabs generated from lab culture

Healthy Chinese horseshoe crabs *T. tridentatus* at the second-instar stage were obtained from Shanghai Ocean University. Total RNA was extracted from the whole horseshoe crabs of six second instars using the TRIzol reagent (Invitrogen, USA), following the manufacturer’s protocol. RNA integrity and quantity were assessed using a NanoDrop spectrophotometer (Thermo Scientific, MA, USA). Paired-end sequencing of the RNA library was performed using the Illumina HiSeq 2500 sequencer (Novogene, Tianjin, China).

### Assembly and filtering for RNA-seq data sets of horseshoe crabs retrieved from the public database

In addition to the RNA-seq data generated from the laboratory culture, a total of 116 publicly available RNA-seq data sets from four horseshoe crab species (17 *L*. *polyphemus*, 12 *C*. *rotundicauda*, 10 *T. gigas*, and 77 *T. tridentatus*) were retrieved from the NCBI SRA repository. The raw reads from these data sets were quality-trimmed and *de novo* assembled using Trinity (version 2.8.5) with default settings (55).

### Virus discovery

To identify potential viral-like contigs in the assembled data sets, the contigs were compared with a locally generated virus database retrieved from NCBI viral protein collection (https://www.ncbi.nlm.nih.gov/genome/viruses) using Diamond BlastX (56). Considering these data sets were from public databases, strict criteria were applied for the identification of putative viruses in each data set. Potential viral contigs with a length greater than 1,000 bp and high identity to seed viral sequences (*e*-value <1 × 10^−20^) were extracted. Thereafter, the identified viral contigs were further compared with the entire NCBI nucleotide and non-redundant protein database to avoid false positive results. To further evaluate the distribution of the discovered viruses across different samples, all 117 data sets were included in the subsequent analysis. Quality-controlled raw reads from each data set were mapped to the corresponding viruses identified within the same horseshoe crab species using Bowtie2 (57) to assess viral abundance across different populations. The accession numbers for all data sets are provided in File S1.

### Viral genome annotation and phylogenetic analysis

Open reading frames (ORFs) of the newly identified viral contigs were predicted using the NCBI ORF Finder tool (https://www.ncbi.nlm.nih.gov/orffinder/) with a minimum length of 100 amino acids (aa). Conserved protein domains were annotated using the NCBI Conserved Domain Search Service (CD-Search) (https://www.ncbi.nlm.nih.gov/Structure/cdd/wrpsb.cgi). To investigate the coverage of the contigs, the adaptor- and quality-trimmed reads from the data sets were mapped back to these viral contigs using Bowtie2 and Samtools (57, 58).

To infer the phylogenetic relationships of the identified viruses, the conserved RdRp regions of these viruses, together with RdRp protein sequences of related reference viruses downloaded from GenBank, were used. The RdRp sequences were aligned using MAFFT with default settings (59), and ambiguously aligned regions were then trimmed using Gblock (60). The best-fit model of aa substitution was selected based on ModelTest-NG (61). Maximum likelihood (ML) trees were estimated using RAxML-NG with 1,000 bootstrap replicates (62). The accession numbers of all sequences used in the phylogenetic analysis are listed in File S2.

### Identification and analysis of nrEVEs in horseshoe crab genomes

The genome assemblies of the four horseshoe crabs, *L. polyphemus*, *C. rotundicauda*, *T. gigas*, and *T. tridentatus* were retrieved from the NCBI genome database with the following accession numbers: GCA_000517525.1 (1.74 GB), GCA_011833715.1 (1.57 GB), GCA_014155125.1 (1.73 GB) and GCA_004210375.1 (2.04 GB), respectively (8, 63–65). A collection of viral protein sequences of newly identified and previously reported horseshoe crab viruses, as well as RNA viruses of the kingdom *Orthornavirae* obtained from the NCBI virus database, was used as queries (File S3) and searched against the genomes of the four horseshoe crab species using the tBlastN algorithm with a cutoff *e*-value of 2 × e^−5^. To eliminate false positives, each identified nrEVE sequence extracted from the genomes accordingly was compared and confirmed with the entire NCBI protein database using a reciprocal BLASTx algorithm. Additionally, to investigate sequence identities among the identified horseshoe crab nrEVEs, alignment of chuviruses glycoprotein-derived EVEs was executed with BioEdit Sequence Alignment Editor (version 7.1.11), and the pairwise distances between nucleotide sequences were performed with the MegAlign program (version 7.1.0) (66, 67).

### Identification of conserved domains associated with transcriptase activity

To explore potential molecular mechanisms underlying hcEVE integration, we extracted the sequences spanning ±10 kb flanking each identified hcEVE in the four horseshoe crab species. These sequences were subsequently analyzed using the NCBI CD-Search tool to identify conserved domains related to reverse transcription, DNA transposition, or other auxiliary functions.

### Experimental confirmation of discovered novel RNA viruses and nrEVEs in *T. tridentatus*

Due to the availability of the horseshoe crab species, experimental confirmation of discovered novel RNA viruses and nrEVEs was conducted in *T. tridentatus*. Briefly, genomic DNA was extracted from *T. tridentatus* using the DNA extraction kit (Promega, USA) following the manufacturer’s instructions. Then, the presence of the identified TtEVEs was confirmed with PCR followed by Sanger sequencing. Additionally, cDNA was synthesized from the extracted total RNA using HiScript II reverse transcription (Vazyme, China), and newly discovered RNA viruses, as well as EVE-containing transcripts of *T. tridentatus,* were confirmed by RT-PCR, subsequently followed by Sanger sequencing. The primers used in this study are listed in Table S5.

### Phylogenetic analysis of nrEVEs of horseshoe crab species

A phylogenetic tree of nrEVEs was constructed based on glycoproteins of nrEVEs and exogenous chuviruses using the method described above. The nrEVE sequences used for this analysis are provided in File S4. For the time-scaled phylogenetic tree of horseshoe crab species, both horseshoe crab genomes and related arthropod species (*Tribolium castaneum* and *Oedothorax gibbosus*) were used for the phylogenetic analysis, and divergence time between species was estimated. Briefly, low-quality sequences were filtered using orthomclFilterFasta with criteria of aa sequence length > 30 and stop codon percentage < 20%. Then, BLASTp was utilized to perform all-vs-all aa similarity analyses with an *e*-value threshold of 1 × e^−5^. Eligible single-copy orthologous genes in each species were identified using OrthoMCL (1:1:1 gene analysis) (68). Multiple alignments were generated by MAFFT, and conserved aa sites were determined with Gblock (60). Using *T. castaneum* as the outgroup, an ML tree was constructed based on the genomes of horseshoe crabs using RAxML with 1,000 bootstrap replicates. The divergence time estimation was performed using MCMCtree and further verified to calibrate the time-tree by the TimeTree program (https://www.timetree.org) (69, 70).

### Analysis of nrEVE transcripts of the four horseshoe crabs

To investigate the transcriptional expression profiles of these nrEVEs across different populations, developmental stages, and tissues of the four horseshoe crab species, 77 representative data sets (17 *L*. *polyphemus*, 12 *C*. *rotundicauda*, 10 *T. gigas*, and 38 *T. tridentatus*) were selected for subsequent analysis. All RNA-seq data sets of *L. polyphemus*, *C. rotundicauda,* and *T. gigas* were chosen, while for *T. tridentatus*, data sets representing different developmental stages were retained, and other biological replicates were removed. Detailed information on these chosen horseshoe crab data sets was provided in Table S4. Quality-controlled raw reads of each data set were mapped to the identified nrEVEs of the corresponding horseshoe crab species with Bowtie2 (57) to explore transcript abundance of nrEVEs in different originated horseshoe crab populations.

To further identify transcripts containing nrEVEs in horseshoe crabs, the assembled contigs from the data sets were searched against a locally customized database comprising all identified horseshoe crab nrEVEs using BlastN. Subsequently, the transcript sequences containing nrEVEs were extracted from the corresponding data set, with the transcript showing the highest coverage and length selected if present in multiple data sets. These sequences were then searched against the genome of the corresponding horseshoe crab species to accurately confirm nrEVE locations within the genome. Moreover, the matched region of EVEs in the horseshoe crab genomes was retrieved and extended by approximately 2,000 bases at both the 5′ and 3′ termini to predict ORFs using the online ORF Finder server (https://www.ncbi.nlm.nih.gov/orffinder). Finally, the abundance of the transcripts was measured using the same method described above.

### sRNA profiles derived from horseshoe crab hcEVEs

To investigate the potential presence of sRNAs derived from hcEVEs, all available sRNA sequencing data from the NCBI SRA were retrieved. The raw sRNA reads were processed using the FASTX-Toolkit v0.0.14 (http://hannonlab.cshl.edu/fastx_toolkit) to remove adapters and low-quality sequences. Clean sRNA reads, ranging from 18 to 30 nucleotides in lengths, were extracted and subsequently mapped to horseshoe crab hcEVEs using Bowtie v1.2.3 with perfect match parameters (71). Downstream analyses were conducted using custom Linux bash scripts.

## Data Availability

Sequences of all novel viruses identified in this study have been deposited in GenBank under accession numbers BK067742–BK067761 and PQ158567–PQ158570. The transcriptome raw reads were deposited in the NCBI Sequence Read Archive (SRA) database under accession number SRX25633775. The data supporting the findings of this study are available in supplemental Tables S1 to S6 and Files S1 and S2.

## SUPPLEMENTAL MATERIAL

Supplemental figures jvi.00164-25-s0001.docx Figures S1 and S2.

Supplemental Excel file jvi.00164-25-s0002.xlsx Files S1 to S4.

Supplemental tables jvi.00164-25-s0003.docx Tables S1 to S6.

ASM does not own the copyrights to Supplemental Material that may be linked to, or accessed through, an article. The authors have granted ASM a non-exclusive, world-wide license to publish the Supplemental Material files. Please contact the corresponding author directly for reuse.
