# Supplementary material for: Diversity and evolutionary history of RNA viruses among different horseshoe crab species
Source: J Virol. 2025 Jun 20;99(7):e00164-25. doi: 10.1128/jvi.00164-25 (PMC12282059; doi:10.1128/jvi.00164-25)
Supplement: Supplemental figures — Figures S1 and S2. [file jvi.00164-25-s0001.docx]

**Supplemental Figure 1**


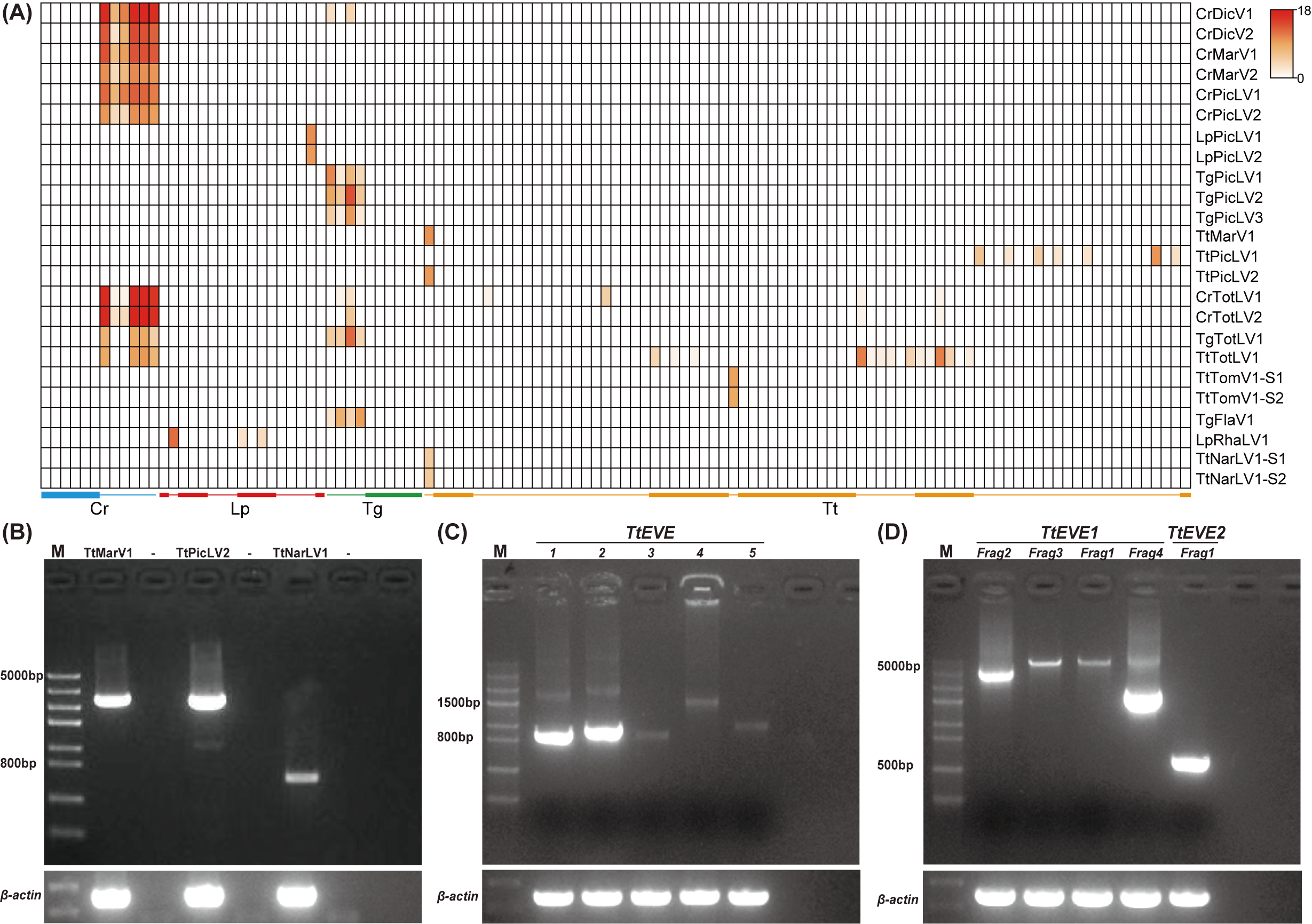


**Supplementary Figure 1. Abundance of newly identified RNA viruses across horseshoe crab populations and experimental validation of viral sequences and hcEVEs in *Tachypleus tridentatus*.** (A) Abundance and distribution of identified RNA viruses across the four horseshoe crab species. The thickness of the lines below represents the datasets contributed by different submitters. Raw data are provided in **Supplemental File S1**. (B) RT-PCR validation of viral contigs identified in *T. tridentatus*. (C) PCR confirmation of genomic integration for TtEVE1, TtEVE2, TtEVE3, TtEVE4, and TtEVE5 in *T. tridentatus*. (D) RT-PCR validation of transcripts derived from TtEVE1 and TtEVE2 in *T. tridentatus*.

**Supplemental Figure 2**


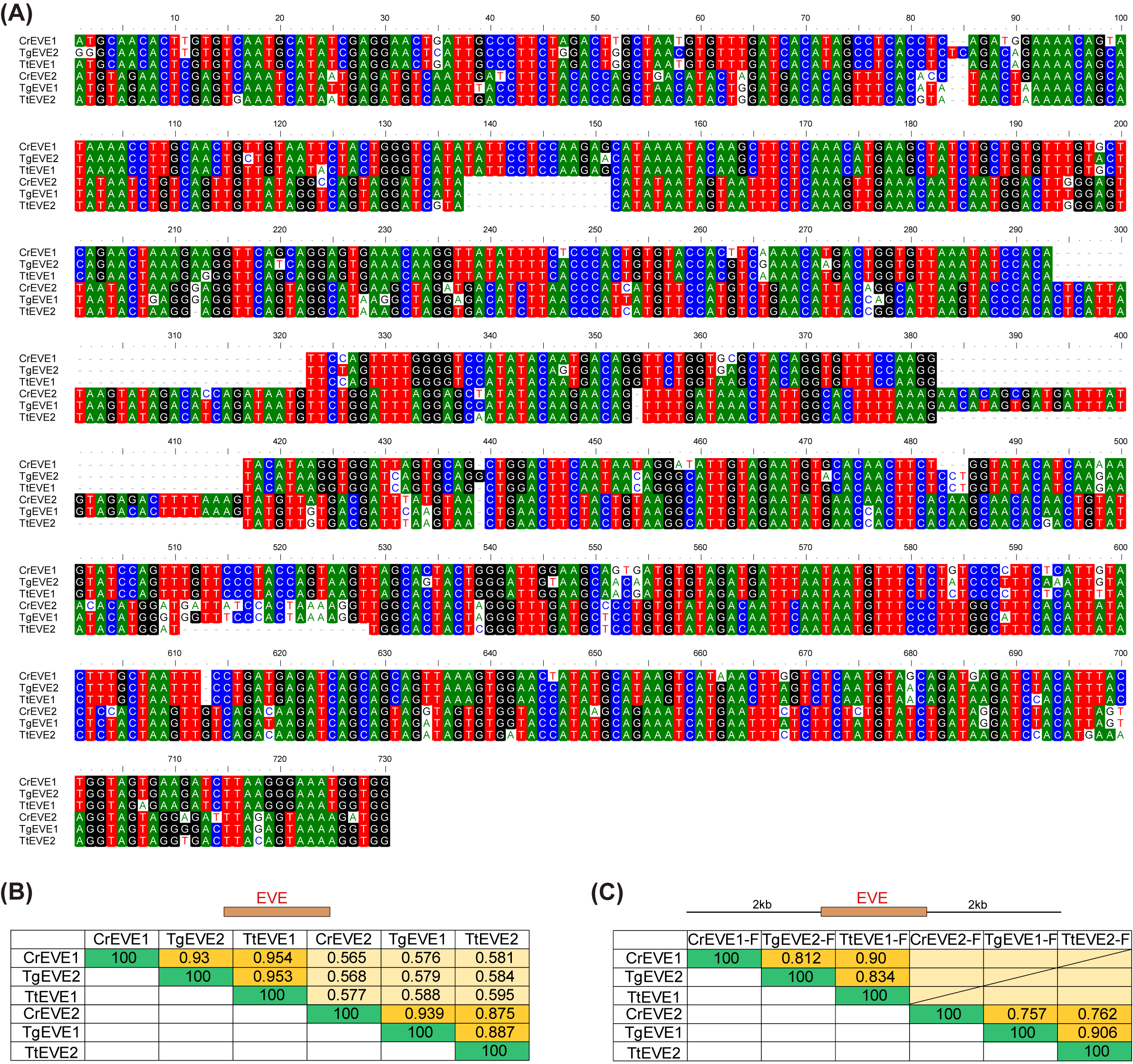


**Supplemental Figure 2. Nucleotide sequence alignment among horseshoe crab nrEVEs sharing homology to the glycoprotein of Tacheng Tick Virus 4.** (A) Alignment of six nrEVEs retrieved from the genomes of *Carcinoscorpius rotundicauda* (CrEVE1 and CrEVE2), *Tachypleus gigas* (TgEVE1 and TgEVE2), and *Tachypleus tridentatus* (TtEVE1 and TtEVE2). (B) The pairwise distances between nucleotide sequences of the six nrEVEs. (C) The pairwise distances between nucleotide sequences of the flanking region of the six nrEVEs.
