# Supplementary material for: Diversity and evolutionary history of RNA viruses among different horseshoe crab species
Source: J Virol. 2025 Jun 20;99(7):e00164-25. doi: 10.1128/jvi.00164-25 (PMC12282059; doi:10.1128/jvi.00164-25)
Supplement: Supplemental tables — Tables S1 to S6. [file jvi.00164-25-s0003.docx]

**Supplemental Table S1. Horseshoe crab datasets derived from public database** **and lab that were infected with RNA viruses.**

| **Accession Numbers** | **Scientific Name** | **University/Institute** | **Locations** | **Tissues** | **Total Bases (Gb)** | **N50 (bp)** |
| --- | --- | --- | --- | --- | --- | --- |
| SRR11233737 | *Tachypleus gigas* | Institute of Molecular and Cell Biology, A*STAR | wild/Singapore | hepatopancreas | 22.49 | 2690 |
| SRR11233738 |  |  | wild/Singapore | gill | 17.55 | 1076 |
| SRR11233735 |  |  | wild/Singapore | muscle | 21.75 | 1462 |
| SRR1241359 | *Tachypleus tridentatus* | National Marine Environment Monitoring Center | Fujian, China | muscle | 4.70 | 2009 |
| SRR7239305 |  | Ocean College, Qinzhou University | Guangxi, China | whole larva | 2.66 | 1557 |
| SRR7630490 |  | Northwestern Polytechnical University | China | hepatopancreas | 4.69 | 1475 |
| SRR30167061 |  | Ningbo University | China | whole body | 6.2 | 1310 |
| SRR5602507 | *Limulus polyphemus* | University of Cambridge | USA | male muscle, heart and liver | 6.83 | 1779 |
| SRR1145732 |  | American Museum of Natural History | USA | walking legs | 7.38 | 2508 |
| SRR6981889 | *Carcinoscorpius rotundicauda* | Institute of Molecular and Cell Biology, A*STAR | Singapore | muscle | 23.60 | 3085 |
| SRR6981892 |  |  |  | egg | 24.56 | 648 |
| SRR6981893 |  |  |  | egg | 21.23 | 2308 |
| SRR6981894 |  |  |  | muscle | 15.46 | 2676 |

**Supplemental Table S2. Novel RNA viruses identified in the four horseshoe crab species.**

| **Tentative virus names (NCBI Accession)** | **NCBI SRR Accession** | **Length**  **(nt)** | **Coverage** | **E-value** | **Homologous virus (genome size, nt)** | **Protein Identities** | **Virus order** | **Virus family** | **Virus Genus** |
| --- | --- | --- | --- | --- | --- | --- | --- | --- | --- |
| Tachypleus gigas picorna-like virus 1 (BK067750) | SRR11233735 | 7,959 | 4,566X | 1.00E-143 | Riboviria sp. (2,712) | 36.55% | *Picornavirales* | Unassigned | Unassigned |
| Tachypleus gigas toti-like virus 1 (BK067755) | SRR11233737 | 6,402 | 19,258X | 0 | Beihai toti-like virus 4 (6,618) | 67.57% | *Ghabrivirales* | *Totiviridae* | Unassigned |
| Tachypleus gigas picorna-like virus 2 (BK067752) |  | 6,434 | 49,828X | 3.00E-156 | Neosmilaster georgianus associated picornavirus 2 (9,384) | 46.57% | *Picornavirales* | Unassigned | Unassigned |
| Tachypleus gigas picorna-like virus 3 (BK067751) |  | 2,014 | 8,988X | 4.00E-153 | Picornaviridae sp. (1,751) | 42.71% | *Picornavirales* | Unassigned | Unassigned |
| Tachypleus gigas flavivirus 1  (BK067760) | SRR11233738 | 10,353 | 1,452X | 0 | Rio Bravo virus (10,140) | 36.10% | *Amarillovirales* | *Flaviviridae* | *Flavivirus* |
| Tachypleus tridentatus tombusvirus 1 (BK067758, BK067759) | SRR1241359 | 2,485 | 3,304X | 0 | Elderberry latent virus (3,892) | 66.76% | *Tolivirales* | *Tombusviridae* | *Pelarspovirus* |
|  |  | 1,027 | 6,420X | 9.00E-162 |  | 64.65% |  |  |  |
| Tachypleus tridentatus toti-like virus 1 (BK067756) | SRR7239305 | 6,598 | 4,909X | 0 | Beihai toti-like virus 4 (6,618) | 71.79% | *Ghabrivirales* | *Totiviridae* | Unassigned |
| Tachypleus tridentatus picorna-like virus 1 (BK067753) | SRR7630490 | 5,658 | 3,703X | 3.00E-138 | Dendrolac virus (8,479) | 28.18% | *Picornavirales* | Unassigned | Unassigned |
| Tachypleus tridentatus marnavirus 1 (PQ158568) | SRR30167061 | 5,393 | 4,501 X | 4e-112 | Picornavirales N_OV_080 (8,584) | 33.74% | *Picornavirales* | *Marnaviridae* | Unassigned |
| Tachypleus tridentatus picorna-like virus 2 (PQ158567) |  | 4,899 | 3,545X | 0 | Aurantiochytrium single-stranded RNA virus 01 (9,035) | 48.97% | *Picornavirales* | Unassigned | Unassigned |
| Tachypleus tridentatus narna-like virus 1 (PQ158569, PQ158570) |  | 1,004 | 806X | 4e-157 | Leptosphaeria biglobosa narnavirus 2 (2,360) | 72.20% | *Wolframvirales* | *Narnaviridae* | Unassigned |
|  |  | 1,043 | 862X | 0 | Leptosphaeria biglobosa narnavirus 2 (2,227) | 77.23% |  |  |  |
| Limulus polyphemus picorna-like virus 1 (BK067748) | SRR5602507 | 9,118 | 3,004X | 4.00E-130 | Wufeng shrew picorna-like virus 16 (3,623) | 36.84% | *Picornavirales* | Unassigned | Unassigned |
| Limulus polyphemus picorna-like virus 2 (BK067749) |  | 5,234 | 3,098X | 9.00E-135 | Wufeng shrew picorna-like virus 5 (9,482) | 40.74% | *Picornavirales* | Unassigned | Unassigned |
| Limulus polyphemus rhabdo-like virus 1 (BK067761) | SRR1145732 | 4,357 | 15,856X | 0 | Rhabdovirus sp. (11,712) | 66.35% | *Mononegavirales* | *Rhabdoviridae* | Unassigned |
| Carcinoscorpius rotundicauda dicistrovirus 1 (BK067744) | SRR6981889 | 9,738 | 54,616X | 0 | Bivalve RNA virus G5 (9,782) | 44.03% | *Picornavirales* | *Dicistroviridae* | Unassigned |
| Carcinoscorpius rotundicauda dicistrovirus 2 (BK067745) | SRR6981892 | 7,270 | 11,451X | 0 | Picornavirales Q_sR_OV_042 (9,939) | 57.29% | *Picornavirales* | *Dicistroviridae* | Unassigned |
| Carcinoscorpius rotundicauda toti-like virus 2 (BK067757) |  | 6,642 | 168,879X | 0 | Beihai toti-like virus 4 (6,618) | 71.53% | *Ghabrivirales* | *Totiviridae* | Unassigned |
| Carcinoscorpius rotundicauda picorna-like virus 1 (BK067746) | SRR6981893 | 7,734 | 13,590X | 0 | Cragig virus 7 (7,881) | 41.12% | *Picornavirales* | Unassigned | Unassigned |
| Carcinoscorpius rotundicauda picorna-like virus 2 (BK067747) |  | 6,866 | 4,010X | 1.00E-161 | Dugejap virus 4 (6,813) | 37.99% | *Picornavirales* | Unassigned | Unassigned |
| Carcinoscorpius rotundicauda marnavirus 2 (BK067743) |  | 6,289 | 2,784X | 0 | Ripithyq virus (7,418) | 48.57% | *Picornavirales* | *Marnaviridae* | Unassigned |
| Carcinoscorpius rotundicauda marna virus 1 (BK067742) | SRR6981894 | 8,994 | 6,338X | 0 | Beihai picorna-like virus 11 (9,245) | 48.30% | *Picornavirales* | *Marnaviridae* | Unassigned |
| Carcinoscorpius rotundicauda toti-like virus 1 (BK067754) |  | 6,941 | 155,429X | 0 | Beihai toti-like virus 4 (6,618) | 69.38% | *Ghabrivirales* | *Totiviridae* | Unassigned |

**Supplemental Table S3. Identification of endogenous viral elements (EVEs) in the genomes of the four horseshoe crab species.**

| **EVE names** | **Length** | **Chr**  **Scaffold** | **Chr position (Orientation)** | **Homologous viruses (host)** | **Protein hits**  **(aa position)** | **Identity** | **E-value** |
| --- | --- | --- | --- | --- | --- | --- | --- |
| Limulus polyphemus EVE1 | 2504 nt | KI894847.1 | 348795-347908 (-) | Tacheng Tick Virus 4 (*Argas miniatus*) | polymerase (1026-1282) | 26.49% | 3.00E-14 |
|  |  |  | 349617-349423 (-) |  | polymerase (748-810) | 38.46% | 6.00E-22 |
|  |  |  | 350233-349658 (-) |  | polymerase (537-724) | 30.14% | 6.00E-22 |
|  |  |  | 350411-350344 (-) |  | polymerase (474-499) | 50.00% | 6.00E-22 |
| Limulus polyphemus EVE2 | 1881 nt | KI901155.1 | 28791-27508 (-) | Herr Frank virus 1 (*Boa constrictor*) | RdRp (876-1352) | 29.56% | 4.00E-68 |
|  |  |  | 29179-28805 (-) |  | RdRp (746-870) | 56.00% | 4.00E-68 |
|  |  |  | 29225-29169 (-) |  | RdRp (731-749) | 52.63% | 4.00E-68 |
|  |  |  | 29388-29221 (-) |  | RdRp (666-732) | 41.79% | 4.00E-68 |
| Limulus polyphemus EVE3 | 538 nt | KI895259.1 | 43504-43379 (-) | Herr Frank virus 1 (*Boa constrictor*) | glycoprotein (433-474) | 35.71% | 4.00E-17 |
|  |  |  | 43758-43552 (-) |  | glycoprotein (346-413) | 52.17% | 4.00E-17 |
|  |  |  | 43916-43758 (-) |  | glycoprotein (291-343) | 32.08% | 4.00E-17 |
| Limulus polyphemus EVE4 | 534 nt | KI895259.1 | 25006-25539 (+) | Herr Frank virus 1 (*Boa constrictor*) | glycoprotein (42-222) | 26.84% | 5.00E-08 |
| Limulus polyphemus EVE5 | 356 nt | KI894713.1 | 25583-25434 (-) | Sanxia atyid shrimp virus 4 (freshwater atyid shrimp) | RdRp (330-379) | 46.00% | 1.00E-12 |
|  |  |  | 25789-25613 (-) |  | RdRp (257-322) | 39.39% | 1.00E-12 |
| Limulus polyphemus EVE6 | 282 nt | KI895339.1 | 38776-38495 (-) | Neuropteran arli-related virus OKIAV105 (*Hemerobius nitidulus*) | RdRp (200-274) | 41.33% | 6.00E-14 |
| Carcinoscorpius rotundicauda EVE1 | 1569 nt | Chr3 | 8248161-8246593 (-) | Tacheng Tick Virus 4 (*Argas miniatus*) | glycoprotein (71-616) | 30.62% | 6.00E-69 |
| Carcinoscorpius rotundicauda EVE2 | 930 nt | Chr16 | 37919851-37918922 (-) | Tacheng Tick Virus 4 (*Argas miniatus*) | glycoprotein (306-610) | 34.07% | 8.00E-39 |
| Carcinoscorpius rotundicauda EVE3 | 480 nt | Chr10 | 9245257-9245012 (-) | Beihai mantis shrimp virus 5 (Mantis shrimp) | hypothetical protein 1 (1175-1256) | 36.59% | 8.00E-08 |
|  |  |  | 9245023-9244778 (-) |  | hypothetical protein 1 (1175-1256) | 36.59% | 8.00E-08 |
| Carcinoscorpius rotundicauda EVE4 | 441 nt | Chr16 | 8478414-8477974 (-) | Neuropteran arli-related virus OKIAV105 (*Hemerobius nitidulus*) | RdRp (247-392) | 30.87% | 9.00E-11 |
| Carcinoscorpius rotundicauda EVE5 | 261 nt | Chr2 | 28740665-28740405 (-) | Mud crab virus (*Scylla serrata*) | non-structural polyprotein (1296-1387) | 32.61% | 2.00E-08 |
| Tachypleus gigas EVE1 | 1634 nt | Chr8 | 111372392-111372234 (-) | Tacheng Tick Virus 4 (*Argas miniatus*) | glycoprotein (551-602) | 35.85% | 5.00E-43 |
|  |  |  | 111372528-111372379 (-) |  | glycoprotein (491-540) | 40.00% | 5.00E-43 |
|  |  |  | 111373102-111372524 (-) |  | glycoprotein (306-487) | 39.38% | 5.00E-43 |
|  |  |  | 111373867-111373286 (-) |  | glycoprotein (96-294) | 28.14% | 1.00E-17 |
| Tachypleus gigas EVE2 | 1624 nt | Chr7 | 127980469-127980933 (+) | Tacheng Tick Virus 4 (*Argas miniatus*) | glycoprotein (72-235) | 24.39% | 3.00E-27 |
|  |  |  | 127980929-127982092 (+) |  | glycoprotein (234-599) | 24.81% | 5.00E-43 |
| Tachypleus gigas EVE3 | 549 nt | Chr1 | 2771319-2771594 (+) | Macrobrachium rosenbergii Taihu virus (*Macrobrachium rosenbergii*) | non-structural polyprotein (1194-1285) | 28.26% | 3.00E-07 |
|  |  |  | 2771592-2771867 (+) |  | non-structural polyprotein (1194-1285) | 28.26% | 3.00E-06 |
| Tachypleus gigas EVE4 | 342 nt | Chr8 | 118637829-118637488 (-) | Beihai mantis shrimp virus 5 (Mantis shrimp) | hypothetical protein 1 (1178-1299) | 32.79% | 2.00E-07 |
| Tachypleus tridentatus EVE1 | 1780 nt | Chr10 | 107041423-107042610 (+) | Tacheng Tick Virus 4 (*Argas miniatus*) | glycoprotein (11-428) | 31.74% | 5.00E-41 |
|  |  |  | 107042108-107043202 (+) |  | glycoprotein (255-637) | 26.02% | 5.00E-41 |
| Tachypleus tridentatus EVE2 | 1013 nt | Chr12 | 107254956-107255285 (-) | Tacheng Tick Virus 4 (*Argas miniatus*) | glycoprotein (422-536) | 37.39% | 8.00E-36 |
|  |  |  | 107255303-107255968 (-) |  | glycoprotein (234-415) | 30.60% | 8E-36 |
| Tachypleus tridentatus EVE3 | 843 nt | Chr9 | 8243291-8243545 (-) | Macrobrachium rosenbergii Taihu virus (*Macrobrachium rosenbergii*) | non-structural polyprotein (1194-1278) | 37.65% | 3.00E-07 |
|  |  |  | 8243345-8243626 (-) |  | non-structural polyprotein (1193-1286) | 36.17% | 8.00E-09 |
|  |  |  | 8243864-8244133 (-) |  | non-structural polyprotein (1194-1283) | 35.56% | 4.00E-07 |
| Tachypleus tridentatus EVE4 | 399 nt | Chr11 | 33047969-33048289 (-) | Macrobrachium rosenbergii Taihu virus (*Macrobrachium rosenbergii*) | non-structural polyprotein (1193-1299) | 33.64% | 2.00E-09 |
|  |  |  | 33048077-33048367 (-) |  | non-structural polyprotein (1193-1289) | 31.96% | 3.00E-06 |
| Tachypleus tridentatus EVE5 | 336 nt | Chr9 | 7630510-7630764 (-) | Macrobrachium rosenbergii Taihu virus (*Macrobrachium rosenbergii*) | non-structural polyprotein (1194-1278) | 37.65% | 1.00E-08 |
|  |  |  | 7630564-7630845 (-) |  | non-structural polyprotein (1193-1286) | 36.17% | 7.00E-10 |

**Supplemental Table S4. Detailed transcriptome information used to identify hcEVE transcripts of horseshoe crabs.**

| **Species** | **Accession Number** | **Total Size (Mb)** | **Submitter** | **Description** |
| --- | --- | --- | --- | --- |
| *Limulus polyphemus* | SRR2616680  SRR3984982  SRR3987665 | 14567.04 | Zhejiang University (ZJU) | *Limulus polyphemus* RNA-seq whole body |
|  | SRR2103466 | 271.17 | University of South Carolina (USC) | Searching for light-interacting genes in transcriptomes from non-model organisms |
|  | SRR2103467 | 267.22 |  |  |
|  | SRR2103465 | 181.08 |  |  |
|  | SRR4215561 | 10413.85 | University of New Hampshire (UNH) | *Limulus polyphemus* Draft Transcriptome and Raw Sequence Reads |
|  | SRR4215560 | 9433.78 |  |  |
|  | SRR4215562 | 8597.57 |  |  |
|  | SRR4215559 | 3891.23 |  |  |
|  | SRR610305 | 131.6 | University of Connecticut (UConn) | *Limulus polyphemus* Transcriptome or Gene expression |
|  | SRR5602507 | 6826.53 | University of Cambridge (CU) | The evolution of small RNA pathways across the arthropods |
|  | SRR5602497 | 3487.2 |  |  |
|  | SRR5602500 | 3419.06 |  |  |
|  | SRR5602501 | 2896.35 |  |  |
|  | SRR1145732 | 7385.42 | American Museum of Natural History (AMNH) | *Limulus polyphemus* Transcriptome or Gene expression |
|  | SRR064415 | 155 | University of Florida - Leonid Moroz (UF-LM) | *Limulus polyphemus* Neuronal Transcriptome |
|  |  |  |  |  |
| *Carcinoscorpius rotundicauda* | SRR14663348 | 4050.39 | Universiti Sains Malaysia (USM) | Delineating the Transcriptome of the Horseshoe Crab, *Carcinoscorpius rotundicauda* |
|  | SRR14663349 | 3394.25 |  |  |
|  | SRR14663350 | 3765.61 |  |  |
|  | SRR14663351 | 3799.18 |  |  |
|  | SRR14663352 | 3579.26 |  |  |
|  | SRR14663353 | 3343.06 |  |  |
|  | SRR6981889 | 23596.63 | Institute of Molecular and Cell Biology, A*STAR (IMCB) | *Carcinoscorpius rotundicauda* transcriptome of muscle2 |
|  | SRR6981890 | 25166.58 |  | *Carcinoscorpius rotundicauda* transcriptome of leg |
|  | SRR6981891 | 20581.9 |  | *Carcinoscorpius rotundicauda* transcriptome of gill |
|  | SRR6981892 | 24558.03 |  | *Carcinoscorpius rotundicauda* transcriptome of egg1 |
|  | SRR6981893 | 21226.58 |  | *Carcinoscorpius rotundicauda* transcriptome of egg2 |
|  | SRR6981894 | 15464.16 |  | *Carcinoscorpius rotundicauda* transcriptome of muscle1 |
|  |  |  |  |  |
| *Tachypleus gigas* | SRR11233737 | 22489.37 | Institute of Molecular and Cell Biology, A*STAR (IMCB) | Coastal horseshoe crab hepatopancreas RNA-seq |
|  | SRR11233735 | 21746.38 |  | Coastal horseshoe crab muscle RNA-seq |
|  | SRR11233736 | 21038.05 |  | Coastal horseshoe crab leg RNA-seq |
|  | SRR11233738 | 17547.84 |  | Coastal horseshoe crab gill RNA-seq |
|  | SRR13900376 | 7546.67 | Universiti Sains Malaysia (USM) | Delineating the Genome, Transcriptome and Proteome of the Horseshoe Crab, *Tachypleus gigas* |
|  | SRR13900377 | 7545.82 |  |  |
|  | SRR13900375 | 6997.6 |  |  |
|  | SRR13900372 | 6875.67 |  |  |
|  | SRR13900374 | 6849.97 |  |  |
|  | SRR13900373 | 6523.73 |  |  |
|  |  |  |  |  |
|  | SRR946952 | 4711.42 | Third Institute of Oceanography (TIO) | *Tachypleus tridentatus* transcriptome |
|  | SRR10845539 | 4333.52 | The Chinese University of Hong Kong (CUHK) | *Tachypleus tridentatus* transcriptome of telson |
|  | SRR10845545 | 4167.45 |  | *Tachypleus tridentatus* transcriptome of blood |
|  | SRR10845541 | 4036.54 |  | *Tachypleus tridentatus* transcriptome of leg |
|  | SRR10845542 | 3875.35 |  | *Tachypleus tridentatus* transcriptome of heart |
|  | SRR10845543 | 3812.12 |  | *Tachypleus tridentatus* transcriptome of chelicerate |
|  | SRR10845544 | 3715.17 |  | *Tachypleus tridentatus* transcriptome of brain |
|  | SRR10845547 | 2937.03 |  | *Tachypleus tridentatus* transcriptome of Tail |
|  | SRR10845538 | 1519.3 |  | *Tachypleus tridentatus* transcriptome of HSC |
|  | SRR18683977 | 2282.71 | Shanghai Ocean University (SHOU) | Transcriptome analysis of *Tachypleus tridentatus* during the molt cycle -Po2 |
|  | SRR18683979 | 2247.14 |  | Transcriptome analysis of *Tachypleus tridentatus* during the molt cycle-in1 |
|  | SRR18683976 | 2246.16 |  | Transcriptome analysis of *Tachypleus tridentatus* during the molt cycle-Po3 |
|  | SRR18683968 | 2240.48 |  | Transcriptome analysis of *Tachypleus tridentatus* during the molt cycle-Po1 |
|  | SRR18683971 | 2215.29 |  | Transcriptome analysis of *Tachypleus tridentatus* during the molt cycle-Ec1 |
|  | SRR18683978 | 2212.01 |  | Transcriptome analysis of *Tachypleus tridentatus* during the molt cycle-in2 |
|  | SRR18683975 | 2206.18 |  | Transcriptome analysis of *Tachypleus tridentatus* during the molt cycle-in3 |
|  | SRR18683969 | 2187.01 |  | Transcriptome analysis of *Tachypleus tridentatus* during the molt cycle-Ec3 |
|  | SRR18683972 | 2160.91 |  | Transcriptome analysis of *Tachypleus tridentatus* during the molt cycle-Pr3 |
| *Tachypleus tridentatus* | SRR18683973 | 2160.37 |  | Transcriptome analysis of *Tachypleus tridentatus* during the molt cycle-Pr2 |
|  | SRR18683970 | 2134.46 |  | Transcriptome analysis of *Tachypleus tridentatus* during the molt cycle-Ec2 |
|  | SRR18683974 | 2033.09 |  | Transcriptome analysis of *Tachypleus tridentatus* during the molt cycle-Pr1 |
|  | SRR7630484 | 5587.79 | Northwestern Polytechnical University (NPU) | *Tachypleus tridentatus* Raw sequence reads of Yellow connective tissue |
|  | SRR7630488 | 4975.39 |  | *Tachypleus tridentatus* Raw sequence reads of Stomach |
|  | SRR7630496 | 4859.16 |  | *Tachypleus tridentatus* Raw sequence reads of Intestine |
|  | SRR7630482 | 4790.77 |  | *Tachypleus tridentatus* Raw sequence reads of Book gill |
|  | SRR7630477 | 4787.02 |  | *Tachypleus tridentatus* Raw sequence reads of Heart |
|  | SRR7630480 | 4768.58 |  | *Tachypleus tridentatus* Raw sequence reads of Muscle |
|  | SRR7630490 | 4690.86 |  | *Tachypleus tridentatus* Raw sequence reads of Hepatopancreas |
|  | SRR7630473 | 4578.98 |  | *Tachypleus tridentatus* Raw sequence reads of Blood |
|  | SRR7630495 | 4347.99 |  | *Tachypleus tridentatus* Raw sequence reads of Compound eyes |
|  | SRR1241359 | 4697.2 | National Marine Environment Monitoring Center (NMEMC) | *Tachypleus tridentatus* transcriptome |
|  | SRR6824222 | 2796.39 | La Trobe University (LTU) | Transcriptome analysis of Chinese horseshoe crab larvae-TaL1_R2 |
|  | SRR6824221 | 2735.94 |  | Transcriptome analysis of Chinese horseshoe crab larvae-TaL1_R1 |
|  | SRR6824224 | 2729.77 |  | Transcriptome analysis of Chinese horseshoe crab larvae-TaL2_R2 |
|  | SRR6824223 | 2677.35 |  | Transcriptome analysis of Chinese horseshoe crab larvae-TaL2_R1 |
|  | SRR6824219 | 2659.95 |  | Transcriptome analysis of Chinese horseshoe crab larvae-TaL3_R1 |
|  | SRR6824220 | 2174.01 |  | Transcriptome analysis of Chinese horseshoe crab larvae-TaL3_R2 |
|  | SRR12023443 | 2721.13 | Huazhong Agricultural University (HSC) | RNA-seq of of *Tachypleus tridentatus* |

**Supplemental Table S5. Primers used in this study.**

| **Primer** | **Sequence (5' to 3')** | **Purpose** |
| --- | --- | --- |
| Tt-*β-actin*-F | AGTGCGACATTGACATCCGT | Amplification of fragment of *β-actin* in *Tachypleus tridentatus* (used as control) |
| Tt-*β-actin*-R | TCCCTGCTCCAGGGTTTCTA |  |
| TtMarV1-F | ATGGAGTGGCTGGAGATCCT | Amplification of viral fragments in *T. tridentatus* |
| TtMarV1-R | TTGTTGCAGTATCCACGCCT |  |
| TtPicLV1-F | AAGGTCGCGTAAGAGTGGTG |  |
| TtPicLV1-R | TGATAAACAGCCCGCCCATT |  |
| TtNarLV1-F | TTGGGAATCCAACAGAGCCC |  |
| TtNarLV1-R | TTGAAGTACACGGTTCGGGG |  |
| TtEVE1-F | CCAGGTCAGTCTGGGGAGAA | Amplification of TtEVE fragments in *T. tridentatus* |
| TtEVE1-R | ACCCACTGTGTACCACGTCA |  |
| TtEVE2-F | ACTGGATGACACAGTTTCACG |  |
| TtEVE2-R | TGCTGGGTCAGCAAAACTCA |  |
| TtEVE3-F | GGGTAGAGCGGGCCTATACT |  |
| TtEVE3-R | TGTGAAGATACAAGCTGTTGTTTTA |  |
| TtEVE4-F | GCCTGTGCAAAGTCCATTCG |  |
| TtEVE4-R | AATCCAATTACGCCCACGGA |  |
| TtEVE5-F | GGGTAGAGCGGGCCTATACT |  |
| TtEVE5-R | TGAGTGGTACACAAGACCTGG |  |
| TtEVE1-Frag2-F | GTTACAGAGGCAGCTGGTGT |  |
| TtEVE1- Frag2-R | CGTTAAGCCAGATGTGGGGA |  |
| TtEVE1- Frag3-F | TCTGAGTCACTCTCAGGGGG |  |
| TtEVE1- Frag3-R | GACCCCAAAACTGGAATGCG |  |
| TtEVE1- Frag1-F | ATGCCTGGAACTTGTAAAAATTCAG | Amplification of TtEVE transcripts in *T. tridentatus* |
| TtEVE1- Frag1-R | TCATACTTGACTGAACTTTAAACC |  |
| TtEVE1- Frag4-F | AACGGGATGCAGTCTAGCAG |  |
| TtEVE1- Frag4-R | TGACTGATGTGGCACTGTGA |  |
| TtEVE2- Frag1-F | TGGTCTACGCCTGAGATTGC |  |
| TtEVE2- Frag1-R | ACTCGGGTTTGATGCTCCTG |  |

**Supplemental Table S6.** **Putative Integration-Related Domains Identified in ±10-kb Flanking Regions of hcEVEs.**

| **EVE name** | **Putative associated domain(s)** | **Accession** | **Domain description** |
| --- | --- | --- | --- |
| Limulus polyphemus EVE1 | DEAD-like_helicase_N super family | cl28899 | N-terminal helicase domain of the DEAD-box helicase superfamily. |
| Limulus polyphemus EVE2 | ps-ssRNAv_RdRp-like super family | cl40470 | conserved catalytic core domain of RNA-dependent RNA polymerase (RdRp). |
|  | PIN_SF super family | cl28905 | PIN (PilT N terminus) domain. |
| Limulus polyphemus EVE3 | DDE_Tnp_1_7 super family | cl16433 | Transposase IS4. |
| Limulus polyphemus EVE4 | RT_RNaseH_2 super family | cl39038 | RNase H-like domain found in reverse transcriptase. |
|  | GIY-YIG_SF super family | cl15257 | GIY-YIG nuclease domain superfamily. |
| Limulus polyphemus EVE5 | DEAD-like_helicase_N super family | cl28899 | N-terminal helicase domain of the DEAD-box helicase superfamily. |
| Limulus polyphemus EVE6 | Mononeg_RNA_pol super family | cl15638 | Mononegavirales RNA dependent RNA polymerase. |
| Carcinoscorpius rotundicauda EVE1 | HTH_48 | pfam17906 | HTH domain in Mos1 transposase. |
|  | Transposase_1 super family | cl47656 | Transposase (partial DDE domain). |
| Carcinoscorpius rotundicauda EVE2 | / | / | / |
| Carcinoscorpius rotundicauda EVE3 | ps-ssRNAv_RdRp-like super family | cl40470 | conserved catalytic core domain of RNA-dependent RNA polymerase (RdRp). |
|  | GIY-YIG_SF super family | cl15257 | GIY-YIG nuclease domain superfamily. |
| Carcinoscorpius rotundicauda EVE4 | Mononeg_RNA_pol super family | cl15638 | Mononegavirales RNA dependent RNA polymerase. |
| Tachypleus gigas EVE1 | / | / | / |
| Tachypleus gigas EVE2 | HTH_48 | pfam17906 | HTH domain in Mos1 transposase. |
|  | Transposase_1 super family | cl47656 | Transposase (partial DDE domain). |
| Tachypleus gigas EVE3 | / | / | / |
| Tachypleus gigas EVE4 | / | / | / |
| Tachypleus tridentatus EVE1 | HTH_48 | pfam17906 | HTH domain in Mos1 transposase. |
|  | Transposase_1 super family | cl47656 | Transposase (partial DDE domain). |
| Tachypleus tridentatus EVE2 | / | / | / |
| Tachypleus tridentatus EVE3 | / | / | / |
| Tachypleus tridentatus EVE4 | / | / | / |
| Tachypleus tridentatus EVE5 | / | / | / |
